# HED-Net: a hybrid ensemble deep learning framework for breast ultrasound image classification

**DOI:** 10.3389/frai.2025.1672488

**Published:** 2026-01-23

**Authors:** Soumya Sara Koshy, L. Jani Anbarasi, Modigari Narendra, Rabindra Kumar Singh

**Affiliations:** School of Computer Science and Engineering, Vellore Institute of Technology, Chennai, India

**Keywords:** breast cancer, classification, ConvNextTiny, convolutional neural network, DenseNet121, EfficientNetB7, ensemble method

## Abstract

**Introduction:**

Breast cancer, one of the most life-threatening diseases that commonly affects women, can be effectively diagnosed using breast ultrasound imaging. A hybrid deep learning based ensemble framework combining the effectiveness of different convolutional neural network models has been proposed for breast ultrasound image classification.

**Methods:**

Three distinct deep learning models, namely, EffcientNetB7, DenseNet121, and ConvNeXtTiny, have been independently trained on breast ultrasound image datasets in parallel to capture complementary representations. Local features are extracted using EffcientNetB7 through depthwise separable convolutions, whereas structural details are preserved by DenseNet121 utilizing dense connectivity. Global spatial relationships are modeled using ConvNeXtTiny via large kernel operations. Diverse local, global, and hierarchical features extracted with respect to multiple perspectives are integrated into a high-dimensional unified representation from which non-linear decision boundaries are learned utilizing XGBoost as the feature fusion classifier. Additionally, a soft voting ensemble method averages the predicted probabilities of the individual convolutional network architectures.

**Results:**

The model was evaluated using the BUSI dataset, the BUS-UCLM dataset, and the UDIAT dataset. The accuracy, precision, recall, F1 score, and AUC values obtained on the BUSI data set are 88.46%, 88.49%, 88.46%, 88.45%, and 95.38%, respectively. On the BUS-UCLM dataset, the corresponding values are 90. 51%, 90. 56%, 90. 51%, 90. 51%, and 96. 23%, respectively. The accuracy, precision, recall, F1 score, and AUC values obtained on the UDIAT dataset are 96.97%, 100.00%, 90.91%, 95.24%, and 99.17%, respectively. The decision-making capability of the model has been highlighted using SHAP and Grad-CAM visualizations, further improving the interpretability and transparency of the model, and making it more robust for breast ultrasound image classification.

**Discussion:**

The HED-Net framework exhibits significant potential for clinical application by enhancing diagnostic accuracy and decreasing interpretation time, particularly in resource-limited environments where expert radiologists are in short supply.

## 1 Introduction

Breast cancer is a deadly disease commonly affecting women, and the complete prevention of breast cancer remains a challenge throughout the world. Breast cancer is a condition in which breast cells grow uncontrollably and become tumors. If left undetected, the tumors can spread throughout the body and become fatal. Breast cancer mortality can be considerably reduced with early detection. Various imaging modalities have been utilized for the diagnosis of breast cancer; the most commonly used one without radiation is ultrasound imaging, in which sound waves are used to capture breast images. Ultrasound imaging is particularly used to examine dense breast tissue (Madjar, 2010). Various computer-aided design techniques have been successfully developed for the detection and classification of breast cancer. Deep learning techniques, which automatically extract features, have been successfully applied in the efficient identification of breast cancer in recent years. Deep learning has become an effective tool for cancer detection and prognosis prediction (Tufail et al., 2021). These technological advancements have not only proven effective in breast cancer detection but have also significantly influenced various medical fields, including reproductive health (Khan et al., 2024), underscoring the transformative potential of artificial intelligence in healthcare.

Existing breast ultrasound image classification methods pose various challenges, including (i) low contrast ultrasound images making it difficult to delineate lesion boundaries and internal structure, (ii) ultrasound images are operator dependent, resulting in uneven image quality, (iii) visual resemblance between benign and malignant images which makes it tedious for even experts to differentiate between the two classes, and (iv) class imbalance in datasets. The interpretability of deep learning models remains a crucial issue. Most of the deep learning models are considered as a black box whose internal workings remain elusive. Socioeconomic factors and family history substantially influence breast cancer awareness and preventive practices, especially in resource-constrained environments (Karmakar et al., 2025). This highlights the significance of creating CAD systems that are versatile and accessible in various clinical contexts.

To address these limitations, a hybrid deep learning based method that combines deep learning and machine learning methods has been proposed for the classification of breast ultrasound images. Three distinct convolutional neural network (CNN) architectures, EfficientNetB7 (Tan and Le, 2019), DenseNet121 (Huang et al., 2017), and ConvNeXtTiny (Liu et al., 2022) have been employed that facilitate the extraction of complementary features from different perspectives, thereby aiding the model to distinguish better between benign, malignant, and normal images. EfficientNetB7 with depthwise convolutions and compound scaling property captures local features and fine-grained patterns. Multi-scale hierarchical features are extracted via dense connections in DenseNet121, whereas with ConvNeXtTiny, global spatial relationships are modeled. The extracted features are fused, which improves discriminative capacity by integrating representations from multiple models. The gradient boosted tree model XGBoost is used for the classification of fused features. XGBoost was selected for feature-level fusion because of its resilience with heterogeneous, high-dimensional data and its impressive clinical efficacy in recent ensemble learning frameworks (Bilal et al., 2024). The soft voting ensemble method is also implemented, in which the predicted probabilities from the three architectures are averaged. The explainable AI is used to untangle the decision-making mechanism of the proposed deep learning model. The proposed model has been made interpretable and transparent by integrating Shapley Additive exPlanations (SHAP) (Lundberg and Lee, 2017) and gradient weighted class activation mapping (Grad-CAM) (Selvaraju et al., 2017). Grad-CAM produces spatial heatmaps that emphasize the areas of the ultrasound image most significant for the prediction. This enables radiologists to ascertain if the network concentrates on clinically significant structures, such as lesion borders, hence enhancing confidence in the classification results. SHAP offers quantitative feature-level attributions, facilitating both global and local comprehension of the contributions of different features to a prediction. Collectively, these methodologies reconcile the disparity between black box predictions and clinical reasoning by correlating model outputs with visual and feature-based evidence, thereby assisting radiologists in verifying AI-assisted diagnoses and enhancing the integration of CAD systems into practical workflows. The significance of model interpretability in clinical environments is underscored by current research in other medical fields. Ahmad et al. (2024)) integrated tree-based SHAP explainable AI into their epileptic seizure detection system, highlighting the increasing agreement that transparent AI decision making is essential for clinical implementation.

The primary contributions of this study are as follows:

- A multiperspective feature extraction approach utilizing three separate CNN architectures to capture feature representations at multiple scales and levels of abstraction.
- The high-dimensional feature fusion is implemented by concatenating deep CNN architectures to generate a unified representation space.
- A gradient-boosted decision tree classifier (XGBoost) is utilized to learn nonlinear relations within the combined feature space.
- A probabilistic ensemble fusion method using soft voting is applied to integrate predictions and enhance generalization across various tumor subtypes.
- Model interpretability is enhanced through Grad-CAM visualizations and SHAP value analysis, ensuring the transparency and clinical trustworthiness of the model.

The remainder of the article is structured as follows. The literature on the classification of breast ultrasound images is reviewed in Section II. The proposed ensemble learning framework for classifying breast ultrasound images is covered in Section III. Experimental results and discussions are presented in Section IV. Section V concludes the article.

## 2 Related works

Several techniques have been put forth to classify ultrasound images of the breast. A deep learning technique using the enhanced ResNet50 was proposed by Gupta et al. (2023)) for classifying breast ultrasound images with a 97.8% accuracy rate. F1 score, precision, and recall obtained were 98.44%, 99.21%, and 97.68%, respectively. This method depends on a single CNN and does not provide interpretability, hence constraining clinical applicability. An ensemble network based on fuzzy ranks has been proposed by Deb and Jha (2023)) for the detection of breast cancer. The model makes use of four distinct base learners in order to benefit from the predictions produced by the base learners DenseNet, VGG-Net, Inception, and Xception. Using the ImageNet dataset, the base learners' initial layer weights are pre-trained. A publicly accessible BUSI is used to refine the last five layers. The final classification is based on the fuzzy rank of the predictions made by the basic learners and obtained an accuracy of 85.23 ± 2.52%. Despite the integration of several learners, the technique exhibits restricted generalization.

Nasiri-Sarvi et al. (2024)) proposed a Mamba-based architecture for classifying ultrasound images of the breast in which the long-range processing power of vision transformers is combined with the inductive bias of a convolutional neural network. The method was evaluated using the BUSI dataset and dataset B with 163 ultrasound images and obtained accuracy values of 95.28 ± 1.89% and 87.50 ± 12.08%, respectively. However, the imbalance in the dataset is not considered, and no visual interpretation of the model has been provided. Alotaibi et al. (2023)) proposed a transfer learning method based on VGG-19, and the model was evaluated using three datasets, namely, KAIMRC with 5693 images (Almazroa et al., 2022), BUSI dataset with 780 images, and Dataset B with 162 images (Yap et al., 2017). A pre-processing scheme with three phases, including RGB fusion, ROI highlighting, and noise filtering utilizing a block matching 3D filtering algorithm, was analyzed, resulting in enhanced classification. The model obtained an accuracy of 87.8% on the BUSI dataset, and the accuracy obtained on the KAIMRC dataset was 85.2%. The model does not integrate ensembling and visualization techniques for model interpretation.

Meng et al. (2024)) presented an ensemble method for the classification of ultrasound images of the breast by integrating a convolutional neural network and a transformer, which optimized the predictions and improved the accuracy. The CNN model was employed to retrieve local features, and global features were extracted using a transformer. The visual interpretations of the predictions are provided using Score CAM. The model is evaluated using the BUSI dataset alone and obtained an F1 score of 98.72% and an accuracy of 98.70%. Jabeen et al. (2022)) suggested a technique that uses transfer learning to train a pre-trained DarkNet-53 model and extracts features from the global average pooling layer. The best features are extracted using the Reformed Gray Wolf (RGW) and Reformed Differential Evaluation (RDE) optimization techniques. The model was evaluated using an augmented BUSI dataset and obtained an accuracy of 99.1%. The model was evaluated using only a single dataset, and no clinical explanation of the model is provided. Yadav et al. (2024)) performed breast ultrasound image classification using modified ResNet-101 and obtained accuracy, precision, F1-score, and recall values of 97.43%, 98.55%, 97.56%, and 96.77%, respectively, on the BUSI dataset. The model is based on a single architecture without ensembling.

Wei et al. (2024)) proposed a Multi Feature Fusion Multi Task model that includes a Contextual Lesion Enhancement Perception (CLEP) module. The model is validated using two publicly accessible datasets, namely, BUSI and MIBUS (Lin et al., 2022), and obtained an accuracy of 95% on the BUSI dataset. The accuracy obtained on the MIBUS dataset was 87.4%, demonstrating generalizability concerns. Ayana et al. (2022)) proposed a multistage transfer learning algorithm where transfer learning from an ImageNet pre-trained model to cancer cell line microscopic images is further used as a pre-trained model for transfer learning on breast cancer ultrasound images. Three pretrained models—ResNet50, InceptionV3, and EfficientNetB2—as well as three optimizers—Stochastic Gradient Descent (SGD), Adagrad, and Adam—were analyzed in the model. The model was tested on two distinct datasets and achieved test accuracies of 99 ± 0.612% and 98.7 ± 1.1% for the ResNet50-Adagrad-based model on the Mendeley dataset and MT-Small-dataset, respectively. An ensemble method utilizing Xception and MobileNet models was proposed by Islam et al. (2024)) for the detection and classification of breast cancer. The method achieved a moderate accuracy of 85.69% on the UDAIT dataset and 87.82% on the BUSI dataset. The gradient class activation technique was utilized to clearly illustrate the model's decision-making process.

Dar and Ganivada (2024)) proposed a method utilizing MobileNet for feature extraction, and the relevant features from the extracted features were selected using a genetic algorithm. An ensemble method that combined decision tree, random forest, gradient boost methods, and K nearest neighbor using a weighted voting scheme was used to classify breast ultrasound images. The method was evaluated using BUSI and UDIAT breast ultrasound image datasets and obtained accuracy values of 96.53% and 97.51%, respectively. However visual interpretation of the model was not provided.

Kalafi et al. (2021)) introduced a technique for classifying breast cancer images that uses an attention module in the modified VGG-16 architecture. An ensemble loss function combining binary cross-entropy with the logarithm of the hyperbolic cosine loss is used in the proposed method. The model was evaluated on a single combined dataset alone. The breast ultrasound Dataset B, with 163 images, is merged with the dataset collected at University Malaya Medical Centre (UMMC) with 276 images. The method obtained an accuracy of 93% and an F1 score of 94%.

An explainable machine learning pipeline for the binary categorization of breast ultrasound images was proposed by Rezazadeh et al. (2022)). This pipeline uses first and second-order features taken from the ultrasound image's region of interest to train an ensemble of decision trees. The proposed method is evaluated using the single BUSI dataset and obtained an accuracy of 91% and an F1 score of 93%.

Moon et al. (2020) proposed a method in which three different convolutional neural network architectures, viz. DenseNet, VGGNet, and ResNet are integrated for the diagnosis of breast cancer and obtained sensitivity, accuracy, specificity, precision, AUC, and F1 score of 92.31%, 94.62%, 95.60%, 90%, 0.9711, and 91.14%, respectively, for the BUSI dataset. The sensitivity, accuracy, specificity, precision, AUC, and F1 score obtained for a private dataset were 85.14%, 91.10%, 95.77%, 94.03%, 0.9697, and 89.36%. An image fusion method was also employed. The private dataset was obtained from Seoul National University Hospital (SNUH, Korean) Ahmad et al., (2022). The visual interpretability of the model was not provided.

Ahmad et al. (2022) proposed a hybrid method utilizing AlexNet and gated recurrent unit (AlexNet-GRU) model for the detection and classification of breast cancer and obtained precision, accuracy, specificity, and sensitivity of 98.10%, 99.50%, 97.50%, and 98.90% on the Pcam dataset. The method was evaluated only on a single dataset without assessing multiclass classification. The latest developments in breast cancer diagnoses have progressively investigated imaging modalities beyond conventional RGB images. Hyperspectral imaging is an innovative image processing approach that addresses the limitations of conventional image processing, which evaluates images across multiple wavelength bands (Leung et al., (2024). Machine learning methods, including Support Vector Machines and Convolutional Neural Networks, can proficiently utilize the enhanced spectral signatures present in Hyperspectral Imaging. Himel et al. (2024a) integrated a generative adversarial network with a feature fusion-based ensemble technique and a weighted average-based ensemble technique for breast ultrasound image classification. The method was evaluated using BUSI, UDIAT, and the Thammasat dataset and obtained an accuracy of 99.7%, F1 score of 99.7%, and AUC score of 99.9%. However, a visual interpretation of the model was not provided.

The various breast ultrasound image classification methods are summarized in Table 1.

**Table 1:** Comparison of breast ultrasound image classification methods.

**References**	**Method used**	**Pros**	**Constraints**
Gupta et al. (2023)	Transfer learning based on ResNet50	High accuracy, feature extraction utilizing residual connections	Single model without ensembling, restricted model interpretability
Deb and Jha (2023)	Fuzzy rank-based ensemble network	Utilizes the features from multiple CNNs, visualization of the decision process	Low performance, Evaluation on a single dataset
Nasiri-Sarvi et al. (2024)	Mamba-based architecture	Captures long-range dependencies, better representation learning, evaluated across multiple datasets	Imbalance in the dataset is not taken into account, no visualization of the decision process
Alotaibi et al. (2023)	Transfer learning based on VGG 19	Extensive pre-processing to enhance model prediction, generalized the model across multiple datasets	Single model without ensembling, No visualization of the model
Meng et al. (2024)	Ensemble model with improved Swin transformer	integrates local and global features, and visualization of the model is included	Evaluation on a single dataset, high complexity
Jabeen et al. (2022)	DarkNet-53-based deep learning and fusion of the best selected features	Feature selection using optimization, high accuracy	Evaluation on a single dataset, no clinical explanation of the model
Yadav et al. (2024)	Modified ResNet-101	Deep residual learning, high accuracy	Single model without ensembling, evaluation on a single dataset
Wei et al. (2024)	Multi-feature fusion multi-task CNN model	Visual interpretation of the model, evaluated using two datasets	decline in performance on MIBUS implies generalizability concerns
Ayana et al. (2022)	Multistage transfer learning with three pretrained models	Two-stage transfer learning, good performance on multiple datasets	Absence of feature level fusion, limited interpretability
Islam et al. (2024)	Ensemble deep CNN model	Integrating the strength of two deep learning models, explainability using Grad-CAM	Moderate accuracy
Dar and Ganivada (2024))	MobileNet, genetic algorithm, and ensemble learning	Ensemble voting, good accuracy	No visualization of the model
Kalafi et al. (2021))	Modified VGG-16 with attention module	Improved feature discrimination utilizing attention mechanism, visual interpretation using Score-CAM	Evaluated on a single dataset
Rezazadeh et al. (2022))	Decision tree ensemble method	Model interpretability	Evaluated on a single dataset, manual extraction of region of interest
Moon et al. (2020))	Ensemble-based CNN architecture with image fusion method	evaluated on multiple datasets	No visual interpretability of the model.
Ahmad et al. (2022))	AlexNet-gated recurrent unit	High accuracy	Evaluated on a single dataset, only binary classification can be done.

## 3 Proposed methodolgy

The proposed HED-Net architecture presents an advanced deep learning architecture for the multiclass classification of breast ultrasound images into benign, malignant, and normal classes via multimodel integration and ensemble methods.

### 3.1 HED-Net architecture

**Figure 1:**
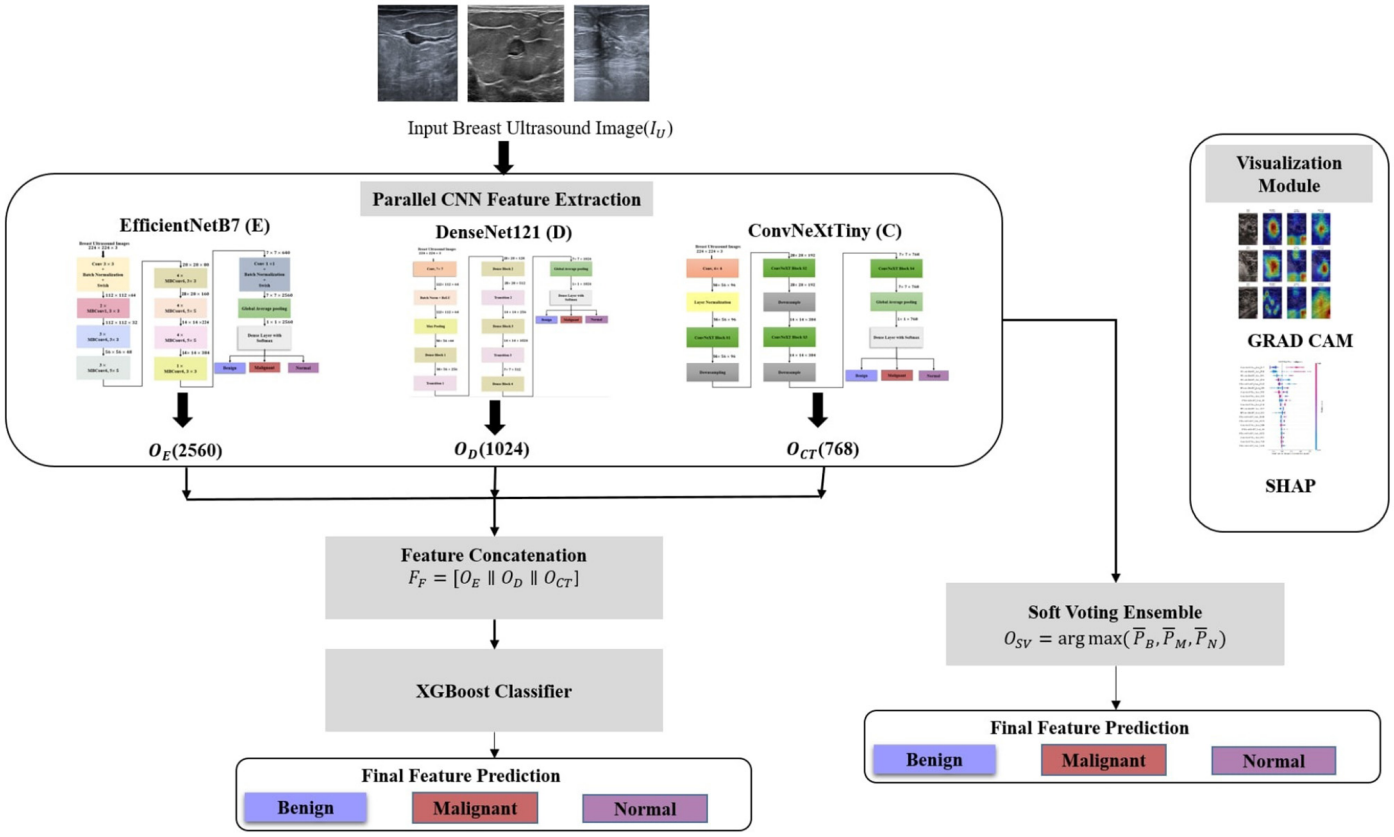
Architecture of HED-Net.

The HED-Net architecture is depicted in Figure 1. The HED-Net architecture for breast ultrasound image segmentation employs three complementary architectures, EfficientNetB7, DenseNet121, and ConvNeXtTiny for extracting hierarchical features from the breast ultrasound images. EfficientNetB7, DenseNet121, and ConvNeXtTiny are used as the foundational networks for our hybrid ensemble learning framework because of their complementary representational abilities. EfficientNetB7 has exhibited exceptional performance in medical image analysis by optimizing accuracy and parameter efficiency using compound scaling. DenseNet121 was selected for its dense connectivity mechanism, which alleviates vanishing gradients and enhances feature reuse, rendering it suitable for capturing intricate structural details in ultrasonic textures. ConvNeXtTiny, a contemporary convolutional architecture influenced by transformer design concepts, provides efficient computing while maintaining robust global feature extraction capabilities, rendering it appropriate for use in real-time or resource-limited settings. All three backbone networks, namely, EfficientNetB7, DenseNet121, and ConvNeXtTiny, were initialized with weights pretrained on ImageNet. This transfer learning methodology is critical due to the small size of medical imaging datasets.

Numerous traditional CNN architectures, including VGG, ResNet, Xception, MobileNet, and Inception, have been extensively employed in medical imaging research. This study specifically concentrated on selecting models that provide complementary and non-redundant feature extraction. Although VGG is simple and extensively utilized, its substantial parameter size renders it inefficient for medical imaging jobs with constrained data. ResNet continues to serve as a robust baseline; nevertheless, its feature representation significantly overlaps with that of DenseNet and EfficientNet, and initial experiments revealed no notable advantage over the chosen models. Xception and Inception emphasize multi-scale feature extraction; however, they may lack the hierarchical detail offered by DenseNet. MobileNet is optimized for efficiency, potentially compromising the representational capacity required for precise lesion classification. The combination of EfficientNetB7 for global semantic features, DenseNet121 for local structural details, and ConvNeXtTiny for efficient global context modeling was anticipated to yield superior feature complementarity compared to the exclusive use of ResNet or VGG.

The stacked feature representation method employed in this study aligns with current medical imaging literature. Himel et al. (2024b)) integrated feature embeddings from two pretrained CNN architectures (EfficientNetB7 and MobileNetV3Large) within a multi-stage network for the detection of acute leukemia, demonstrating that multi-branch feature fusion enhances classification performance across diverse datasets. Recent research indicates that integrating information from several convolutional pipelines markedly enhances classification performance in both medical and non-medical image domains. Himel and Hasan (2025)) introduced a width-scaled lightweight architecture incorporating nested feature fusion and channel spatial attention for the recognition of Bengali sign language, attaining state-of-the-art outcomes using stacked feature representations. Their methodology underscores the advantages of concurrent convolutional operations and multiple-stage fusion, reinforcing the rationale for incorporating EfficientNetB7, DenseNet121, and ConvNeXtTiny into a cohesive ensemble framework.

The input ultrasound images (*I*_*U*_) are fed to the three parallel deep learning architectures. Fine-grained textures and homogenous patterns are detected by EfficientNetB7. It processes the *I*_*U*_ with compound scaled convolutional blocks enhanced by squeeze and excitation to extract the feature map *O*_*E*_ as shown in Equation (1).


$$\begin{array}{l} O_{E}=F_{E}\left(I_{U}\right) \end{array}$$ (1)

DenseNet121 maintains edge continuity for smooth boundaries and speculated margins. Hierarchical features (*O*_*D*_) are extracted from *I*_*U*_ via dense patterns as shown in Equation (2).


$$\begin{array}{l} O_{D}=F_{D}\left(I_{U}\right) \end{array}$$ (2)

ConvNeXtTiny employs advanced CNN architectures integrated with transformer-inspired elements to extract *O*_*CT*_ from *I*_*U*_ as given in Equation (3).


$$\begin{array}{l} O_{CT}=F_{CT}\left(I_{U}\right) \end{array}$$ (3)

The *O*_*E*_, *O*_*D*_, and *O*_*CT*_ extracted from the global average pooling layer of each of the CNNs are concatenated to form a unified feature representation *F*_*F*_ as shown in Equation (4).


$$\begin{array}{l} F_{F}=\left[O_{E}∥O_{D}∥O_{CT}\right] \end{array}$$ (4)

The concatenated features are fed to XGBoost for classification, which classifies the input ultrasound images into benign, malignant, and normal. Dominant modalities from each CNN architecture are identified via feature importance analysis. The soft voting ensemble method has also been implemented by combining the predictions of the individual convolutional neural network models by averaging their predicted probabilities.

### 3.2 Feature extraction

Each of the convolutional neural network architectures is trained separately on the *I*_*U*_ to learn the hierarchical feature representations from input data. *O*_*E*_, *O*_*D*_, and *O*_*CT*_ are extracted from the global average pooling layer or pre-classification layer of each model since it provides the most semantically rich and high-level discriminative features suitable for fusion. Local texture patterns, as well as global structural information, which are essential for accurately classifying breast lesions, have been captured.

#### 3.2.1 EfficientNetB7

EfficientNetB7 is a convolutional neural network architecture starting with a convolutional layer (C) followed by batch normalization (BN) and Swish activation (S), transforming breast ultrasound images into a feature map of dimension (112 × 112 × 64) as shown in Equation (5).


$$\begin{array}{l} Z_{O}=S\left(\text{BN}\left(C_{3\times 3}\left(X\right)\right)\right) \end{array}$$ (5)

A series of mobile inverted bottleneck blocks is incorporated with the expansion phase (*Z*_*E*_), depth-wise separable convolutions (*Z*_*DW*_), and squeeze and excitation modules (*Z*_*S*_*E*__) to enhance channel-wise recalibration. These components are efficacious for capturing precise differences in lesion morphology for the classification of breast ultrasound images. The expansion convolutions are given in Equation (6),


$$\begin{array}{l} Z_{E}=S\left(\text{BN}\left(C_{1\times 1}\left(Z_{O}\right)\right)\right) \end{array}$$ (6)

Depthwise(*Z*_*DW*_) convolutions are given in Equation (7), where DC represents depth-wise convolutions.


$$\begin{array}{l} Z_{DW}=S\left(\text{BN}\left(DC_{3\times 3\text{ or }5\times 5}\left(Z_{E}\right)\right)\right) \end{array}$$ (7)

Squeeze and excitation function (*Z*_*S*_*E*__) is defined in Equation (8).


$$\begin{array}{l} Z_{S_{E}}=\sigma \left(w_{2}R\left(w_{1}G\left(Z_{DW}\right)\right)\right)⊙Z_{DW} \end{array}$$ (8)

where σ is the sigmoid activation function, *w*_1_ and *w*_2_ are the learnable weight matrices, R represents the ReLU activation function, G represents global average pooling, and ⊙ denotes element-wise multiplication. Channels are reduced to the output dimension via projection as in Equation (9).


$$\begin{array}{l} z_{\text{out}}=\text{BN}\left(C_{1\times 1}\left(Z_{S_{E}}\right)\right) \end{array}$$ (9)

The model attains optimal performance by scaling its depth (d), width (w), and input resolution (r), by utilizing a compound scaling coefficient, as given in Equation (10).


$$\begin{array}{l} d=\alpha^{\emptyset }w=\beta^{\emptyset }r=\gamma^{\emptyset } \end{array}$$ (10)

For EfficientNetB7 the values are α = 1.2, β = 1.1, γ = 1.15 and ∅ = 7. The final stage of the model includes a 7 × 7 × 640 convolutional output followed by global average pooling(G) and 1 × 1 convolution for projecting the features into a 2560-dimensional space as shown in Equation (11).


$$\begin{array}{l} O_{E}=C_{1\times 1}\left(G\left(F_{\text{CONV}}\right)\right)\in {\mathbb{R}}^{2560} \end{array}$$ (11)

**Figure 2:**
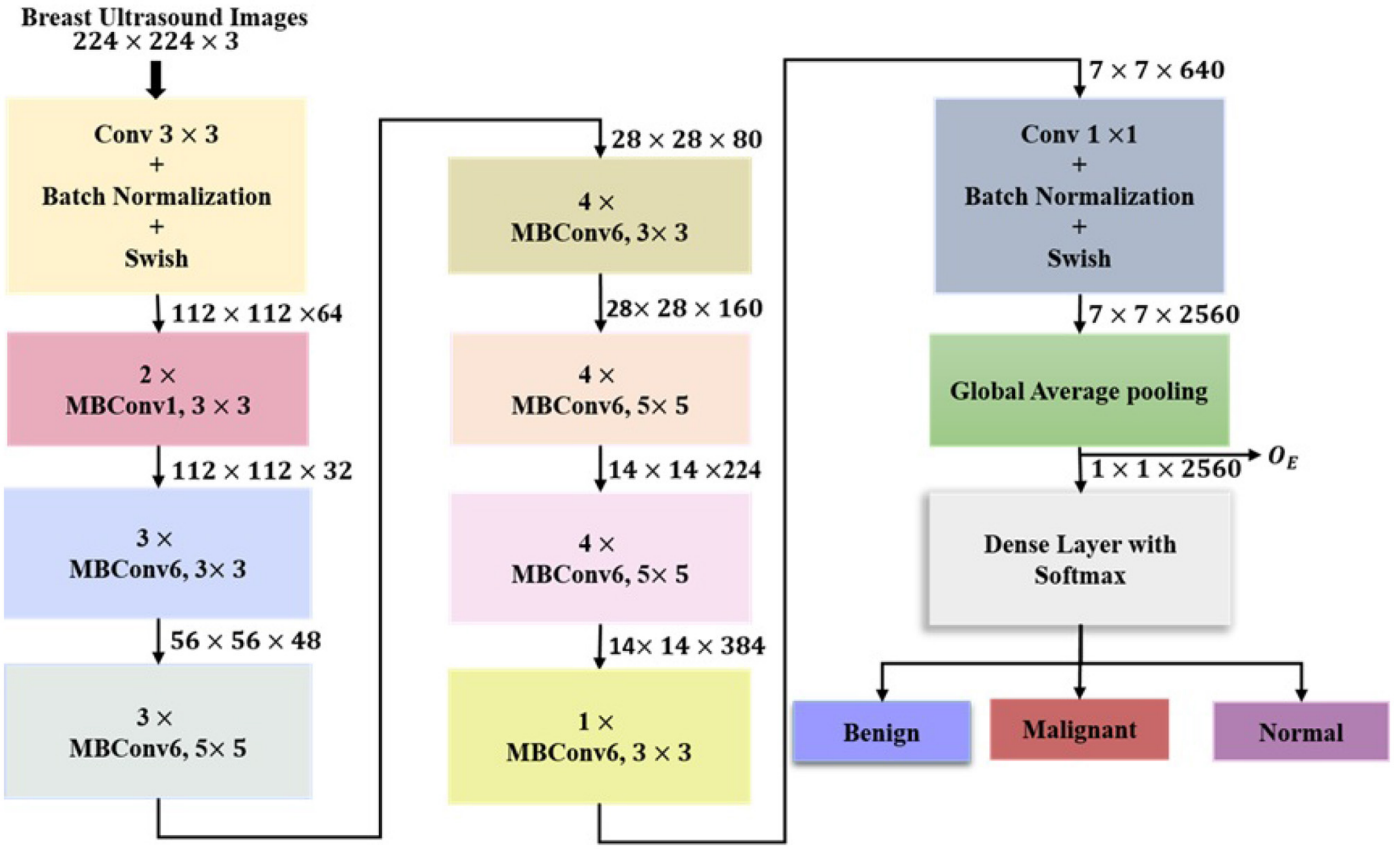
Architecture of EfficientNetB7.

The feature vector from the global average pooling layer (*O*_*E*_) is extracted for concatenation, before the application of the softmax layer, since it contains high-level semantic representations of the input image. The diverse feature learning of the model is preserved by utilizing the pre-final feature vector of dimension 2560 as shown in Equation (11). The architecture of the EfficientNetB7 is shown in Figure 2.

#### 3.2.2 DenseNet121

DenseNet121 is a deep learning architecture with dense connectivity, where each layer within a dense block receives inputs from all the preceding layers, strengthening gradient flow and promoting efficient feature reuse. Breast ultrasound images of dimension 224 × 224 × 3 initially undergo feature extraction via 7 × 7 convolutional layer followed by batch (BN), ReLU activation (R), and 3 × 3 max pooling (*M*_3 × 3_) as shown in Equation (12).


$$\begin{array}{l} F_{DI}=M_{3\times 3}\left(R\left(\text{BN}\left(C_{7\times 7}\left(X\right)\right)\right)\right) \end{array}$$ (12)

The feature maps generated are processed through four dense blocks containing 6, 12, 24, and 16 convolutional layers, respectively. The output feature map of the *i*^*th*^ layer in the current dense block is generated by applying the composite function H to the feature maps from the previous layers 0 to *i*−1 in the same dense block, as shown in Equation (13).


$$\begin{array}{l} D_{i}=H_{i}\left(\left[D_{0},D_{1},\ldots ,D_{i-1}\right]\right) \end{array}$$ (13)

The composite function *H*_*i*_ is given in Equation (14).


$$\begin{array}{l} H_{i}=C_{3\times 3}\left(R\left(\text{BN}\left(C_{1\times 1}\left(R\left(\text{BN}\left(Y\right)\right)\right)\right)\right)\right) \end{array}$$ (14)

Y represents the concatenation of all the feature maps from earlier layers in the dense block. Each dense block is followed by a transition layer consisting of 1 × 1 convolutions and average pooling (*A*_2 × 2_), which performs downsampling and channel compression. The functioning of the *i*^*th*^ transition layer is given in Equation (15).


$$\begin{array}{l} T_{i}=A_{2\times 2}\left(C_{1\times 1}\left(R\left(\text{BN}\left(D_{i}\right)\right)\right)\right) \end{array}$$ (15)

The mid-level spatial and edge information is captured by DenseNet121, improving the generalization capability of the classification model. The final feature map 7 × 7 × 1024 from dense block 4 (*D*_4_) is reduced to a 1024 dimensional vector via global average pooling(G) as shown in Equation (16).


$$\begin{array}{l} O_{D}=G\left(D_{4}\right)\in {\mathbb{R}}^{1024} \end{array}$$ (16)

**Figure 3:**
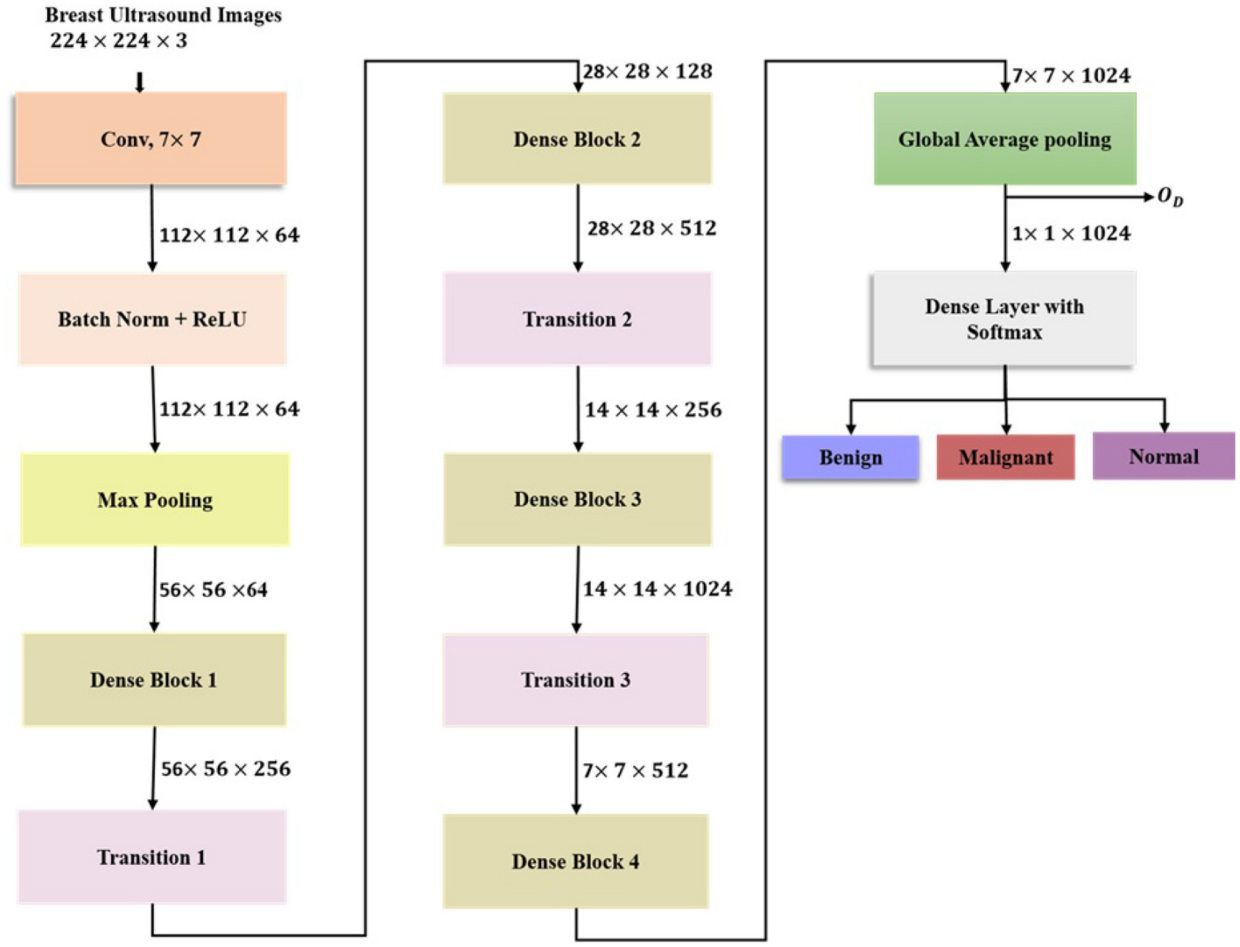
Architecture of DenseNet121.

This feature vector is further concatenated with the features extracted from the EfficientNetB7 and ConvNeXtTiny to form a feature representation for breast ultrasound image classification. The DenseNet121 assures significant feature reuse, alleviates vanishing gradients, and improves interpretability via hierarchical feature integration. The architecture of DenseNet121 is given in Figure 3.

#### 3.2.3 ConvNeXtTiny

ConvNeXtTiny is a transformer design inspired convolutional neural network architecture with an initial 4 × 4 convolutional layer, which transforms the input images of dimension 224 × 224 × 3 into non-overlapping patches of dimension 56 × 56 with 96 channels. Layer normalization is applied to reduce internal covariance shift and to control feature scaling across channels, thereby facilitating quick convergence. Four stages of convNeXt blocks with intermediate downsampling layers follow layer normalization (O(LN)).

Each ConvNeXT block includes depthwise convolutions (DC), layer normalizations (LN), GeLU activations (G), channel compression, and dropout. The output of each block is fed back to the input (I(CN)) via residual connections. The functioning of each ConvNeXT block (*O*(*CN*_*i*_)is shown in Equation (17).


$$\begin{array}{l} O\left(CN_{i}\right)=\left(DC_{1\times 1}\left(G\left(L\left(C_{3\times 3}\left(O\left(LN\right)\right)\right)\right)\right)\right)+I\left(CN_{i}\right) \end{array}$$ (17)

The first, second, and fourth block consists of three convNeXt blocks each, and the third stage contains nine blocks. The channel dimensions of the four stages are 96, 192, 384, and 768, respectively, which are progressively doubled via downsampling, which includes layer normalizations and 2 × 2 convolutions as shown in Equation (18).


$$\begin{array}{l} F_{DS}=C_{2\times 2}\left(L\left(DS_{\text{in}}\right)\right) \end{array}$$ (18)

The 7 × 7 × 768 feature map from the fourth ConvNeXT block(*CN*_4_) is subjected to global average pooling to extract a compact representation of tumor characteristics as shown in Equation (20).


$$\begin{array}{l} O_{CT}=G\left(CN_{4}\right)\in {\mathbb{R}}^{768} \end{array}$$ (19)

**Figure 4:**
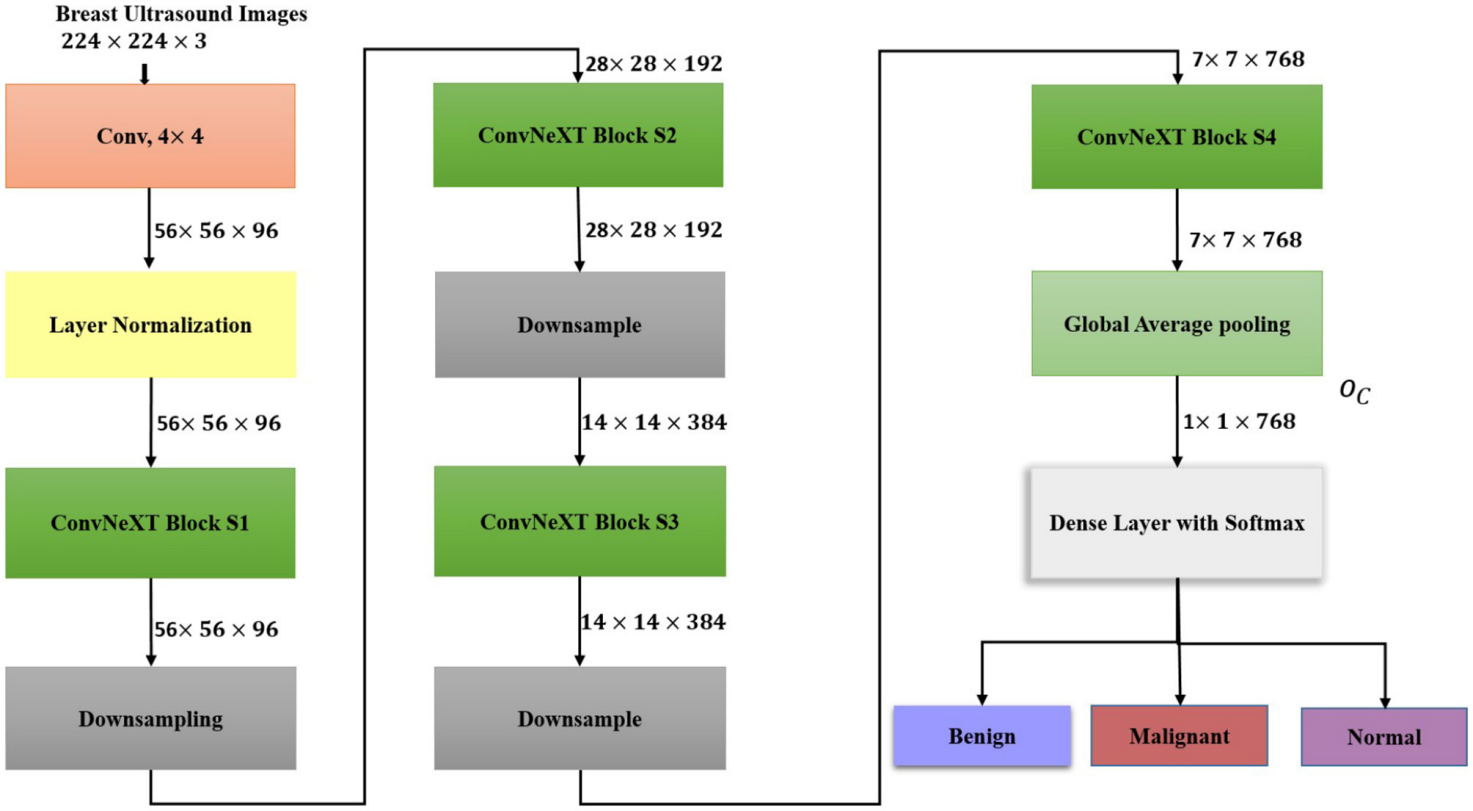
Architecture of ConvNeXtTiny.

The output feature vector from EfficientNetB7 (*O*_*E*_) is fused with feature vectors from DenseNet121 (*O*_*D*_) and ConVNeXtTiny (*O*_*C*_*T*__) to form a unified feature representation for beast ultrasound image classification. The architecture of ConvNeXtTiny is shown in Figure 4.

### 3.3 XGBoost ensemble method

The fused feature vector of dimension 4,352 generated by combining the feature vectors from the three CNN architectures is fed to the gradient boosted decision tree learner (XGBoost) (Liew et al., 2021), to learn complex decision boundaries for lesion classification, as given in Equation (20).


$$\begin{array}{l} ŷ=\text{XGBoost}\left(F_{F}\right) \end{array}$$ (20)

ŷ represents the class probability score for the benign, malignant, and normal categories.

XGBoost is an ensemble method that constructs a chain of decision trees with each successive tree correcting the errors of its predecessors. The decision trees are built sequentially, and the output of all the previous trees is combined additively as shown in Equation (21),


$$\begin{array}{l} {ŷ}_{i}=\overset{K}{\underset{k=1}{\sum }}f_{k}\left(x_{i}\right) \end{array}$$ (21)

where ŷ_*i*_ is the final predicted value, *f*_*k*_(*x*_*i*_) is the output of the *k*^*th*^ tree, and K represents the number of trees. Trees are constructed iteratively to minimize the error from previous trees. The objective function for the XGBoost classifier is given in Equation (22).


$$\begin{array}{l} O\left(\theta \right)=l\left(y_{i},{ŷ}_{i}\right)+\overset{K}{\underset{k=1}{\sum }}\Omega \left(f_{k}\right) \end{array}$$ (22)

where *l*(*y*_*i*_, ŷ_*i*_) is the categorical cross entropy loss function calculating the difference between actual value and predicted value, Ω(*f*_*k*_) is the regularization term for reducing complex trees by penalizing the number and size of leaves in the tree as given in Equation (23).


$$\begin{array}{l} \Omega \left(f\right)=\gamma T+\frac{1}{2}\lambda \overset{T}{\underset{j=1}{\sum }}w_{j}^{2} \end{array}$$ (23)

where γ is the regularization parameter for controlling the complexity of the tree, and T is the number of leaves in the tree. The squared weight of the leaves $w_{j}^{2}$ is penalized by the parameter λ. XGBoost uses the second Taylor expansion for efficient optimization as in Equation (24).


$$\begin{array}{l} L^{\left(t\right)}\approx \overset{n}{\underset{i=1}{\sum }}\left[g_{i}f_{t}\left(x_{i}\right)+\frac{1}{2}h_{i}f_{t}^{2}\left(x_{i}\right)\right]+\Omega \left(f_{k}\right) \end{array}$$ (24)

where *g*_*i*_ and *h*_*i*_ are the first (gradient) and second (Hessian) derivatives of the loss function. At each node, the best split is determined by calculating the information gain as given by Equation (25).


$$\begin{array}{l} \text{Information Gain}=\frac{1}{2}\left[\frac{G_{L}^{2}}{H_{L}+\lambda }+\frac{G_{R}^{2}}{H_{R}+\lambda }-\frac{{\left(G_{L}+G_{R}\right)}^{2}}{H_{L}+H_{R}+\lambda }\right]-\gamma \end{array}$$ (25)

where *G*_*L*_, *G*_*R*_ are sums of gradients in left and right child nodes, and *H*_*L*_, *H*_*R*_ are sums of Hessians in left and right child nodes. The XGBoost classifier makes final predictions based on the concatenated features, and the feature importance is visualized to understand which of the features from the various architectures contribute most significantly to the model's decision process.

### 3.4 Soft voting ensemble method

The soft voting aggregates the class probabilities predicted by the EfficientNetB7, DenseNet121, and ConvNeXtTiny to generate the final decision. Soft voting is an ensemble method in which the probability scores for each of the base models for all the classes are considered, and the weighted average of these probabilities is computed to derive the final prediction.

The probabilities predicted by each of the models, EfficientNetB7, DenseNet121, and ConvNeXtTiny, for the three classes B(benign), M(malignant), and N(normal) are averaged across the three models. Let $p_{E}^{B}$, $p_{E}^{M}$, and $p_{E}^{N}$ represent the probabilities predicted by EfficientNetB7 for the benign, malignant, and normal classes, respectively. The class with the maximum average probability is selected as the maximum prediction. Similarly, let $p_{D}^{B}$, $p_{D}^{M}$, and $p_{D}^{N}$ denote the class probabilities predicted by DenseNet121, and $p_{C}^{B}$, $p_{C}^{M}$, and $p_{C}^{N}$ be the class probabilities predicted by ConvNeXtTiny. The average probability for each class B (${\bar{P}}_{B}$), M(${\bar{P}}_{M}$) and N(${\bar{P}}_{N}$) across the three models are calculated as given in Equations (26), Equation (27), and Equation (28).


$$\begin{array}{l} {\bar{P}}_{B}=\frac{p_{E}^{B}+p_{D}^{B}+p_{C}^{B}}{3} \end{array}$$ (26)

$$\begin{array}{l} {\bar{P}}_{M}=\frac{p_{E}^{M}+p_{D}^{M}+p_{C}^{M}}{3} \end{array}$$ (27)

$$\begin{array}{l} {\bar{P}}_{N}=\frac{p_{E}^{N}+p_{D}^{N}+p_{C}^{N}}{3} \end{array}$$ (28)

The output prediction is given as the class with the maximum average probability as given by Equation (29).


$$\begin{array}{l} O_{\text{SV}}=\arg\max\left({\bar{P}}_{B},{\bar{P}}_{M},{\bar{P}}_{N}\right) \end{array}$$ (29)

The soft voting ensemble method integrates the advantages of multiple models by averaging their probabilistic predictions, yielding more dependable and interpretable classifications for breast ultrasound images.

The overall algorithm of the model is given in Algorithm 1.

Algorithm 1 HED-Net: hybrid ensemble classification framework. 
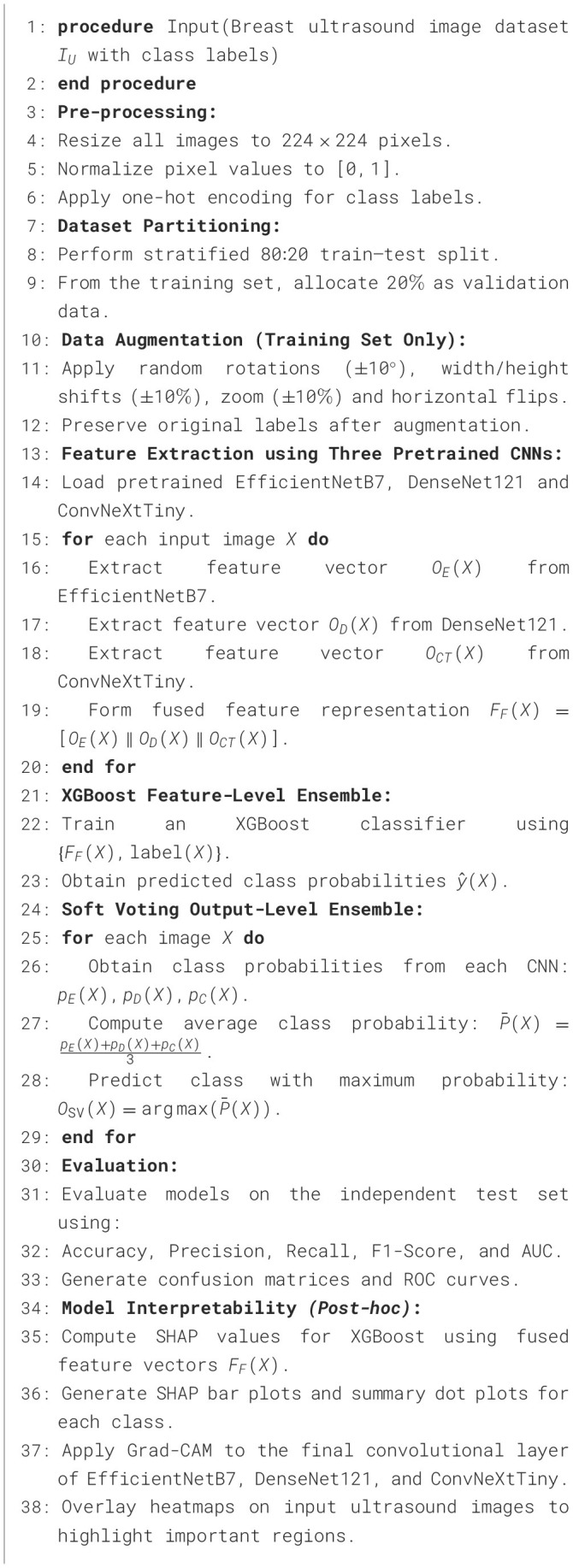

## 4 Experimental results

In this section, the dataset used is described along with the data pre-processing techniques. Training and testing criteria, along with the data augmentation methods, are also explained.

### 4.1 Dataset description

Three breast ultrasound image datasets have been used to evaluate the proposed method. With 780 images divided into benign, malignant, and normal groups, the Breast Ultrasound Image Dataset (BUSI) (Al-Dhabyani et al., 2020) is the first dataset used. The Breast Ultrasound Lesion Segmentation dataset (BUS-UCLM) (Vallez et al., 2025), which consists of 683 images categorized into benign, malignant, and normal classes, is also used to assess the approach. The third dataset used to assess the model is the UDIAT dataset (Yap et al., 2017) with 163 ultrasound images. The benign class involves breast images with masses or lumps that are not cancerous, whereas the malignant class involves images with masses that are cancerous and can spread outside the breast, eventually affecting the whole body. The normal class consists of non-cancerous images without masses or lumps.

#### 4.1.1 Breast Ultrasound Image dataset (BUSI)

**Figure 5:**
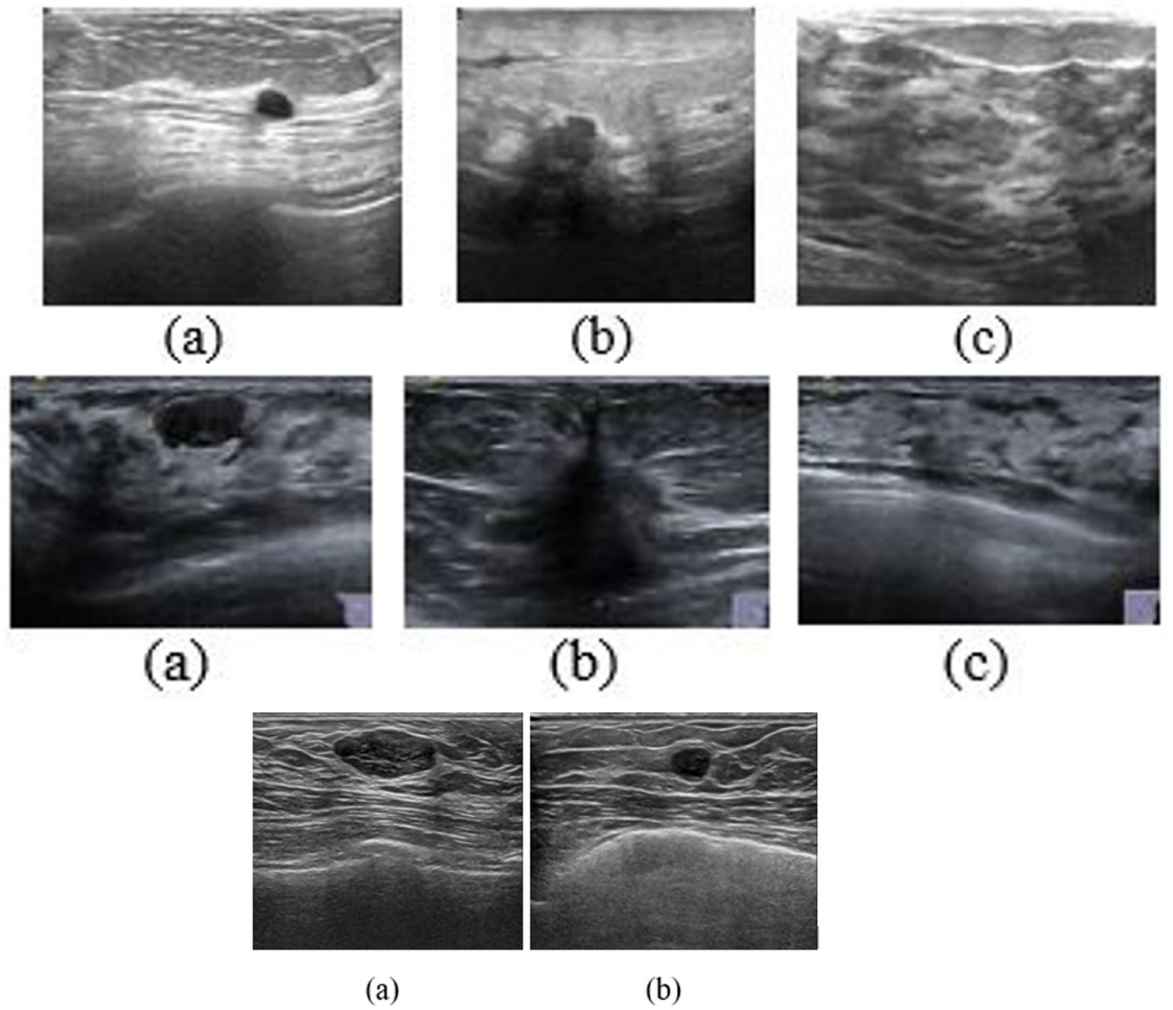
Images from three datasets **(a)** Benign, **(b)** Malignant, **(c)** Normal. **(Top)** BUSI, **(Middle)** BUS-UCLM, and **(Bottom)** UDIAT.

The Breast Ultrasound Image dataset (BUSI) has been used to evaluate the model. The dataset comprises 780 ultrasound images obtained from 600 female patients, categorized into benign, malignant, and normal classes. The dataset includes original as well as ground truth images. This public dataset is extensively utilized for research on breast lesion classification and segmentation. The top row of the Figure 5 shows sample benign, malignant, and normal images from the BUSI dataset.

#### 4.1.2 BUS-UCLM dataset

The BUS-UCLM dataset includes 683 images from 38 patients, of which 174 images are benign, 90 are malignant, and 419 are normal. Ultrasound scans were acquired from 2022 to 2023, utilizing the Siemens Acuson S2000 ultrasound system. Multiple images were obtained for each patient, captured from distinct breast cross sections to guarantee thorough coverage of the area of interest. The ground truth is provided as RGB segmentation masks in separate files, where black denotes normal breast tissue, green signifies benign tumors, and red represents malignant lesions. The segmentation annotations given by expert radiologists facilitate precise model training and assessment, rendering this dataset a significant resource in the field of computer vision and public health, enabling the development of models for distinguishing between benign and malignant tumors in breast ultrasound images. The sample images from the BUS-UCLM dataset are shown in the middle row of the Figure 5.

#### 4.1.3 UDIAT dataset

UDIAT dataset consists of 163 ultrasound images acquired using the Siemens ACUSON Sequoia C512 system 17L5 HD linear array transducer (8.5 MHz) from the UDIAT Diagnostic Centre of the Parc Tauli Corporation, Sabadell (Spain) in 2012. Out of the 163 cancerous images, 109 were benign images, and 54 were normal images. The sample images from the UDIAT dataset are shown at the bottom of the Figure 5.

### 4.2 Experimental setup

Google Colab notebooks, a cloud computing environment, were used to complete the suggested job. For creating a deep neural learning model, Google Colab provides a graphics processing unit (GPU) and a tensor processing unit (TPU). This made it easier for the deep learning model to be trained and run effectively. Table 2 shows the system configuration utilized for the experimentation of the proposed HED-Net.

**Table 2:** Computer Configuration of the HED-Net.

**Item**	**Configuration**
Processor	Intel (R) Xeon (R) Gold 6230 CPU @2.10GHz
Graphics card	NVIDIA Quadro RTX 5000 16 GB
Ram size	64 GB
Hard-disk size	2 TB

### 4.3 Data pre-processing and augmentations

The images of the BUSI dataset are resized to 224 × 224 to match the size of the convolutional neural networks. The image pixels are normalized to the range of 0–1. One-hot encoding is applied to convert class labels to categorical classes for multiclass classification.

The BUS-UCLM dataset comprises 683 images along with their ground truth RGB segmentation masks. The black masks indicate normal breast tissues, green masks indicate benign lesions, and the red masks indicate malignant lesions. The labels are extracted from the masks based on color detection and are encoded using one-hot encoding for multiclass classification.

Each of the BUSI, BUS-UCLM, and UDIAT datasets has been split into training, validation, and testing subsets. A preliminary 80:20 stratified split was employed to acquire a blind test set consisting of 156 images for BUSI, 137 images for BUS-UCLM, and 33 images for UDIAT. Twenty percent of the remaining training subset was allocated as a validation set to optimize model hyperparameters and implement early stopping.

Various transformations have been applied to the images in the BUSI and BUS-UCLM dataset to artificially improve their size. The issue of class imbalance, frequently observed in medical datasets, was addressed by the implementation of data augmentation techniques (Haider et al., 2025). The methods used for data augmentation include random rotations of up to 10 degrees, width and height shifts of up to 10%, zoom variations of up to 10%, and horizontal flips. The original label information of the images is maintained to ensure consistency of the class assignments. Additionally, nearest neighbor filling is used to address any gaps introduced during augmentation. These modifications significantly enhance the diversity of the training data, allowing the model to generalize better to variations in lesion scale, orientation, and position. Table 3 shows the dataset partitioning for training, test, and validation subsets.

**Table 3:** Dataset partitioning for BUSI, BUS-UCLM, and UDIAT datasets.

**Dataset**	**Partition**	**Total**	**Benign**	**Malignant**	**Normal**
BUSI	Original	780	437	210	133
	Training	499	280	134	85
	Validation	125	70	34	21
	Test	156	87	42	27
BUS-UCLM	Original	683	174	90	419
	Training	436	111	57	268
	Validation	110	28	15	67
	Test	137	35	18	84
UDIAT	Original	163	109	54	N/A
	Training	104	70	34	N/A
	Validation	26	17	9	N/A
	Test	33	22	11	N/A

Values indicate the number of samples per class for training, validation, and testing subsets.

Training samples for BUSI, BUS-UCLM, and UDIAT datasets are shown in Table 4. Augmentation is implemented on-the-fly exclusively on the training set; the validation and test sets remain unchanged.

**Table 4:** Training samples before and after data augmentation for each dataset.

**Dataset**	**Original training samples**	**Augmentation factor**	**Approx. augmented samples**
BUSI	499	15 ×	≈7, 485
BUS-UCLM	436	15 ×	≈6, 540
UDIAT	104	10 ×	≈1, 040

**Figure 6:**
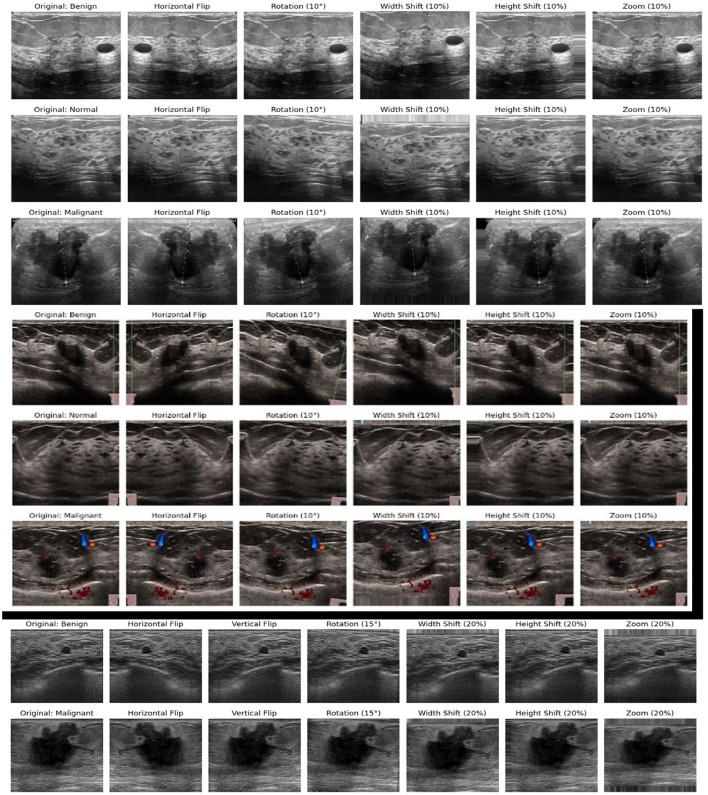
Data augmentation on BUSI, BUS-UCLM, and UDIAT datasets. **(Top three)** BUSI, (Middle three) BUS-UCLM, and **(Bottom two)** UDIAT.

The top three rows of Figure 6 demonstrate the data augmentation techniques applied to benign, malignant, and normal images in the BUSI dataset; the middle three rows of the Figure 6 show the results of the data augmentation in the BUS – UCLM dataset. The data augmentation in the UDIAT dataset are shown at the bottom of the Figure 6.

### 4.4 Hyperparameter tuning

Table 5 illustrates the hyperparameters used during the training of the deep neural networks and the XGBoost classifier. Deep learning models were optimized with a learning rate of 1 × 10^−4^, a batch size of 16, and early stopping combined with learning rate decrease callbacks to mitigate overfitting. All CNN models were trained via the Adam optimizer.

**Table 5:** Hyperparameters for the proposed HED-Net model.

**Model(s)**	**Hyperparameter**	**Value**
EfficientNetB7, DenseNet121, ConvNeXtTiny	Learning rate	1 × 10^−4^
	Batch size	16
	Epochs	15
	Dropout rate	0.30
XGBoost	n_estimators	100
	learning_rate	0.10
	max_depth	6
	eval_metric	mlogloss

**Figure 7:**
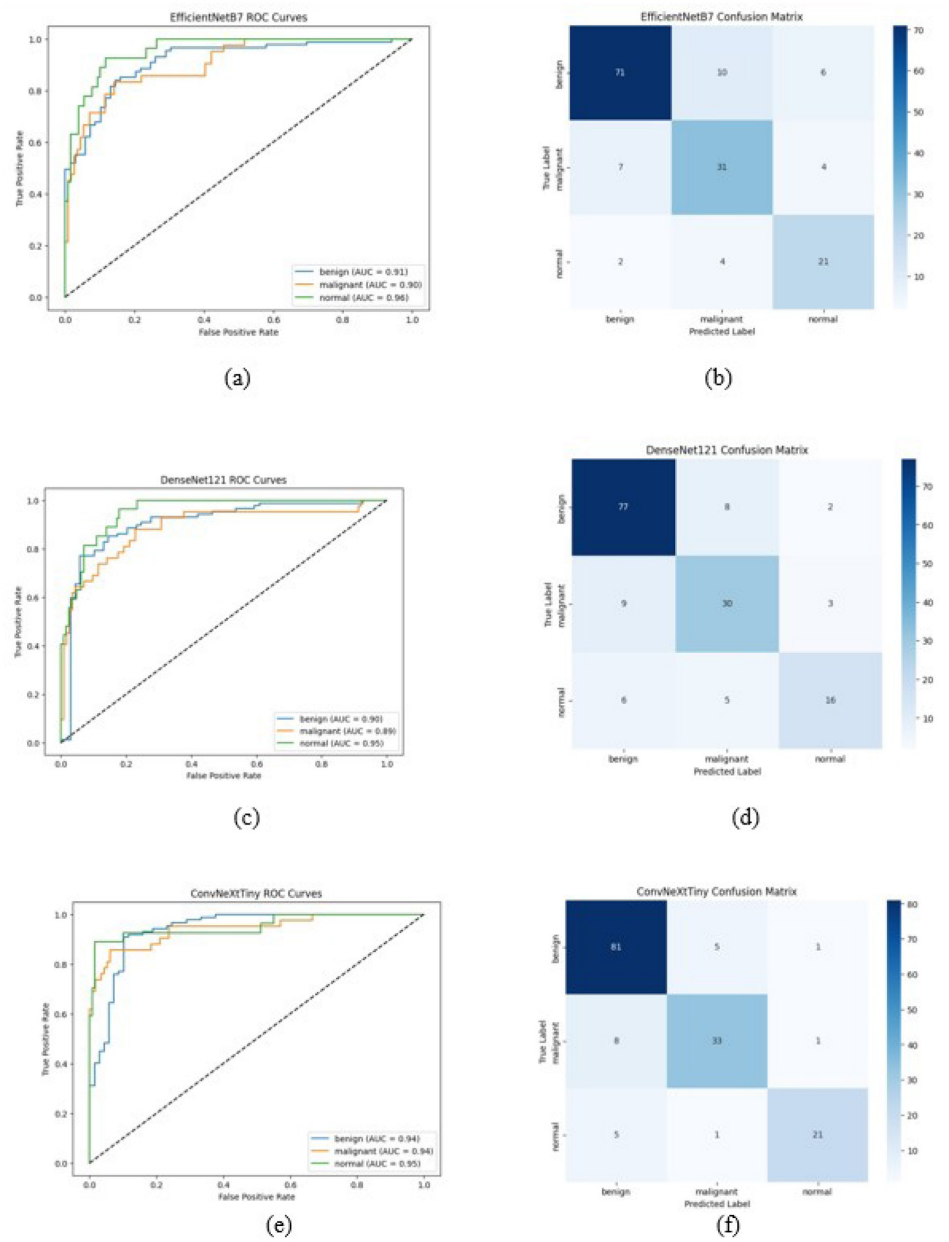
ROC Curve and Confusion Matrix on BUSI dataset **(a, b)** EfficientNetB7, **(c, d)** DenseNet121, and **(e, f)** ConvNeXtTiny in the classification of breast ultrasound images into benign, malignant, and normal categories. The x-axis denotes the false positive rate, while the y-axis signifies the true positive rate. Each colored line represents a distinct class: benign (blue), malignant (orange), and normal (green), with corresponding Area Under the Curve values. The diagonal dashed line signifies the random classifier baseline (AUC = 0.5) (Test set size = 156).

**Figure 8:**
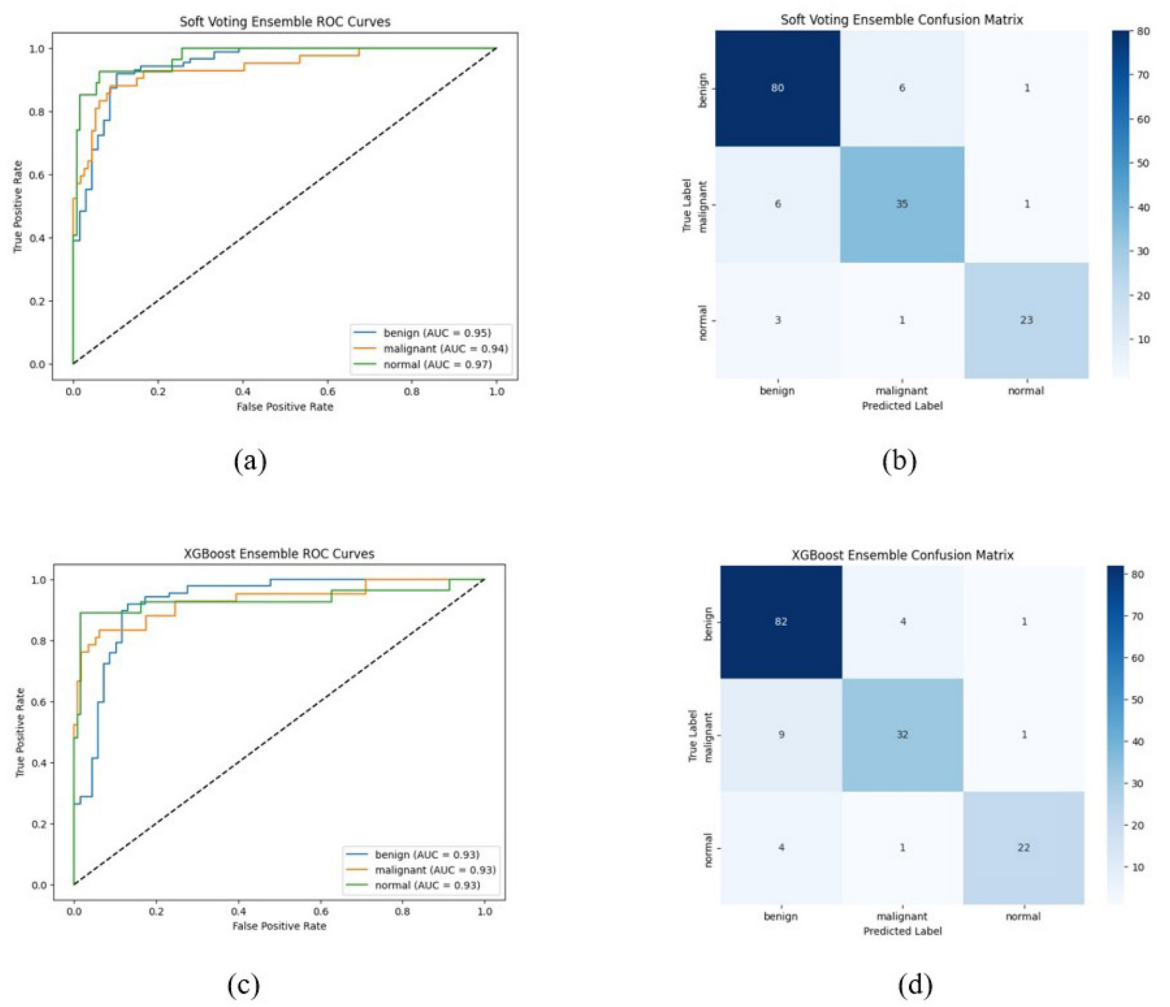
ROC curve and confusion matrix on BUSI dataset **(a, b)** XGBoost, **(c, d)** soft voting ensemble (Test set size = 156).

The ROC curve and confusion matrix obtained for EfficientNetB7, DenseNet121, and ConvNeXtTiny on the BUSI dataset are shown in Figure 7. The ROC curve and confusion matrix obtained for XGBoost and Soft Voting ensemble on the BUSI dataset are shown in Figure 8.

#### 4.4.1 Evaluation metrics

Various metrics used to evaluate the model include accuracy, precision, recall, and F1-score. Accuracy is the ratio of the correctly classified predictions to the total number of predictions made by the model, as given in Equation (30).


$$\begin{array}{c} \text{Accuracy}=\frac{TP+TN}{TP+TN+FP+FN} \end{array}$$ (30)

Precision is the ratio of the model's true positive classifications to the total positive classifications made by the model, as given by Equation (31).


$$\begin{array}{c} \text{Precision}=\frac{TP}{TP+FP} \end{array}$$ (31)

Recall or sensitivity is the ratio of actual positive predictions that are correctly identified, as given by Equation (32).


$$\begin{array}{c} \text{Recall}=\frac{TP}{TP+FN} \end{array}$$ (32)

F1 score is the harmonic mean of precision and recall as given in Equation (33).


$$\begin{array}{c} \text{F1 Score}=\frac{2\times \text{Precision}\times \text{Recall}}{\text{Precision}+\text{Recall}} \end{array}$$ (33)

### 4.5 Performance evaluation

Breast ultrasound images are analyzed separately utilizing three different CNN architectures (EfficientNetB7, DenseNet121, and ConvNeXtTiny), subsequently employing two ensemble methodologies (feature-level fusion with XGBoost and soft voting). Comprehensive metric values for each model and dataset are presented in Tables 6–8.

**Table 6:** Performance analysis of the various models on BUSI dataset.

**Model**	**Accuracy (%)**	**Precision (%)**	**Recall (%)**	**F1 Score (%)**	**AUC (%)**
EfficientNetB7	78.85	79.77	78.85	79.14	92.56
DenseNet121	78.85	78.65	78.85	78.52	91.39
ConvNeXtTiny	86.54	86.64	86.54	86.39	94.55
XGBoost ensemble	87.18	87.29	87.18	87.00	93.01
Soft voting ensemble	**88.46**	**88.49**	**88.46**	**88.45**	**95.38**

Bold values indicate the best performing results for each evaluation metric among the compared models.

**Table 7:** Performance analysis of the various models on BUS-UCLM dataset.

**Model**	**Accuracy (%)**	**Precision**	**Recall (%)**	**F1 Score (%)**	**AUC (%)**
EfficientNetB7	85.40	89.09	85.40	86.01	95.86
DenseNet121	78.83	78.37	78.83	78.26	90.22
ConvNeXtTiny	87.59	88.74	87.59	87.60	96.70
XGBoost ensemble	86.13	86.26	86.13	85.76	93.64
Soft voting ensemble	**90.51**	**90.56**	**90.51**	**90.51**	**97.23**

Bold values indicate the best performing results for each evaluation metric among the compared models.

**Table 8:** Performance analysis of the various models on UDIAT dataset.

**Model**	**Accuracy (%)**	**Precision (%)**	**Recall (%)**	**F1 Score (%)**	**AUC (%)**
EfficientNetB7	90.91	100	72.73	84.21	96.69
DenseNet121	90.91	90	81.82	85.71	96.69
ConvNeXtTiny	87.88	88.89	72.73	80	94.21
XGBoost ensemble	84.85	100	54.55	70.59	86.36
Soft voting ensemble	**96.97**	**100**	**90.91**	**95.24**	**99.17**

Bold values indicate the best performing results for each evaluation metric among the compared models.

The optimal individual backbone on the BUSI dataset is ConvNeXtTiny. The soft voting ensemble enhances classification accuracy by approximately 2.2% (from 86.54% to 88.46%) and results in a relative AUC increase of about 0.9% compared to the optimal individual AUC. The feature-level XGBoost ensemble surpasses the individual CNNs; however, soft voting is the most precise configuration on BUSI.

In the BUS-UCLM dataset, soft voting consistently surpasses all individual backbones, yielding a relative accuracy enhancement of approximately 3.3% in comparison to the highest-performing singular model (ConvNeXtTiny). The AUC increases by approximately 0.6% compared to the most robust single-network baseline, indicating that the probabilistic aggregation of the three models produces better-calibrated predictions on this more heterogeneous dataset.

The impact of ensembling is significantly more evident on the UDIAT dataset. Soft voting yields an approximate 6.7% enhancement in accuracy compared to the optimal individual backbone and increases AUC by roughly 2.6%. Although the XGBoost ensemble does not surpass the individual models in accuracy on UDIAT, it is valuable for assessing feature importance and enhances the output-level ensemble.

**Figure 9:**
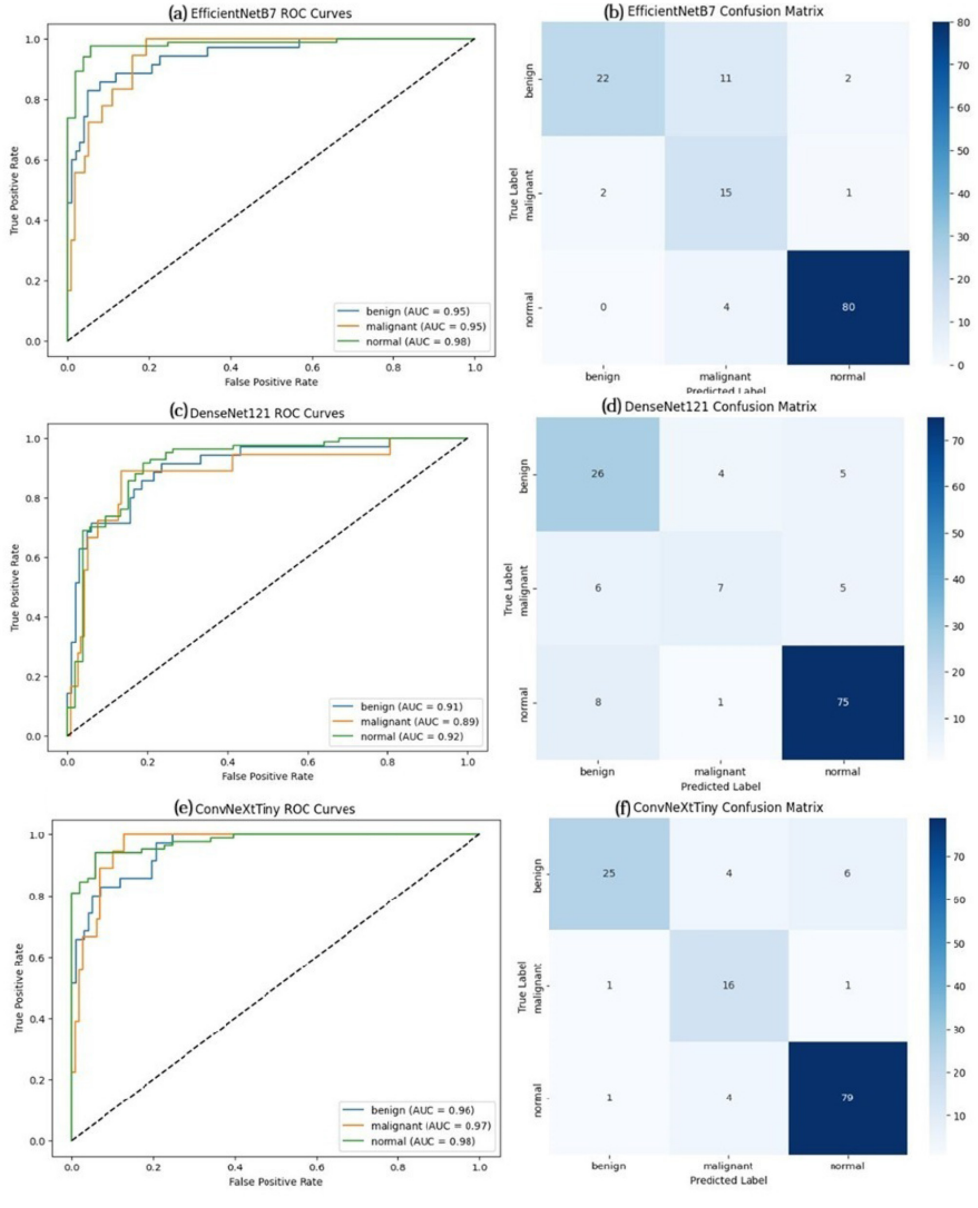
ROC curve and confusion matrix on BUS-UCLM dataset **(a, b)** EfficientNetB7, **(c, d)** DenseNet121, and **(e, f)** ConvNeXtTiny (Test set size = 137).

**Figure 10:**
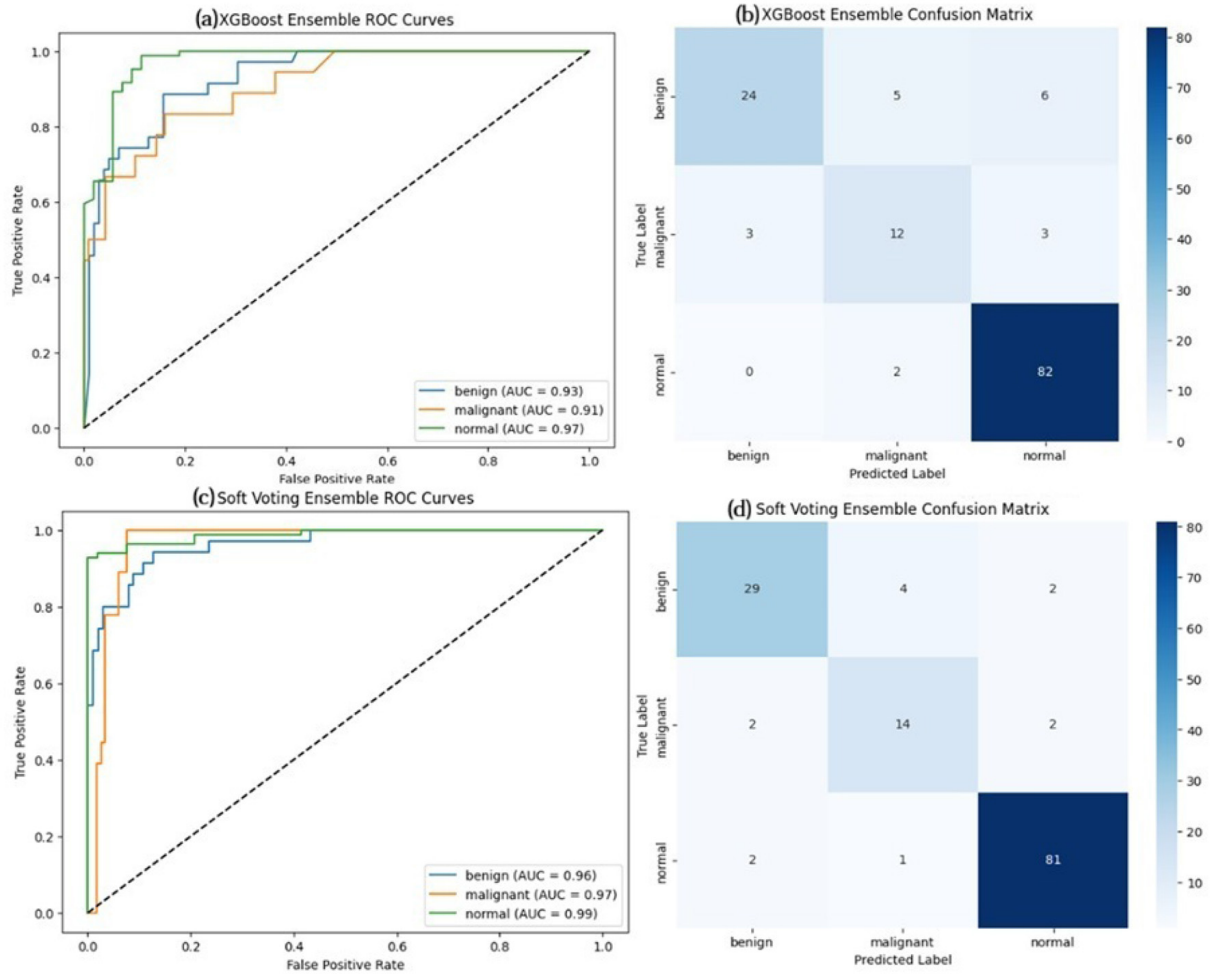
ROC curve and confusion matrix on BUS-UCLM dataset **(a, b)** XGBoost, **(c, d)** soft voting ensemble (Test set size = 137).

**Figure 11:**
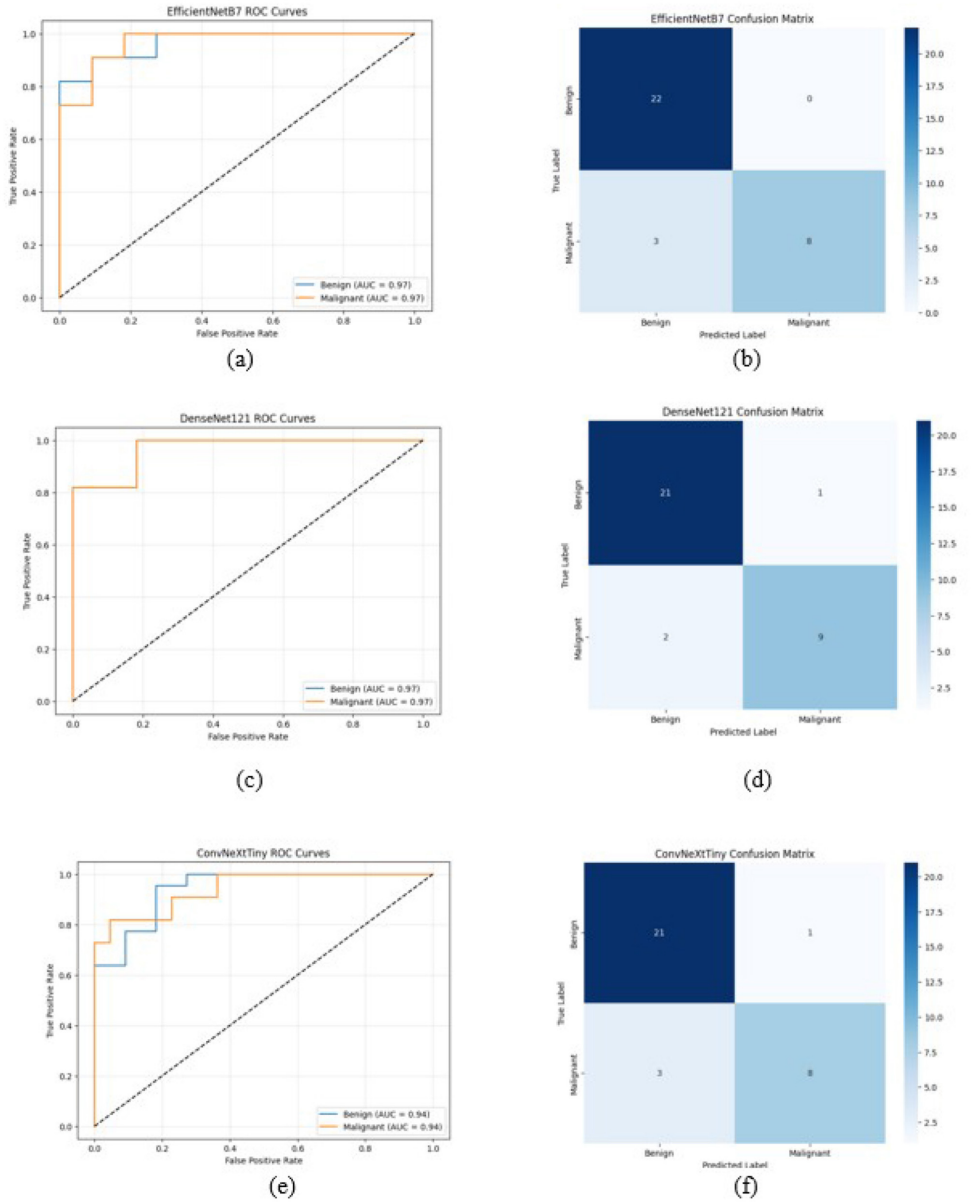
ROC curve and confusion matrix on UDIAT dataset **(a, b)** EfficientNetB7, **(c, d)** DenseNet121, and **(e, f)** ConvNeXtTiny (Total test set size = 33).

**Figure 12:**
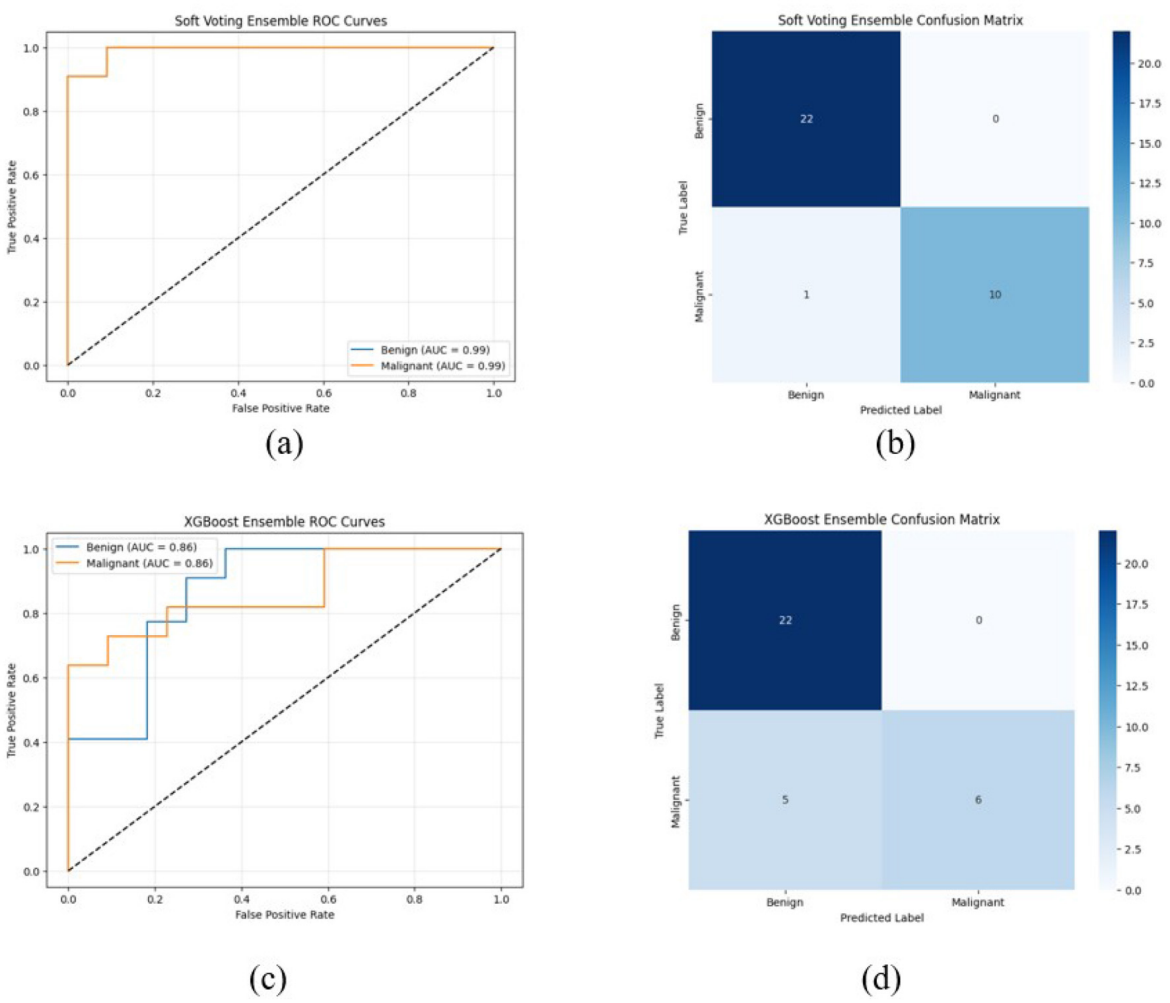
ROC curve and confusion matrix on UDIAT dataset **(a, b)** XGBoost, **(c, d)** soft voting ensemble (Total test set size = 33).

In all three datasets, the proposed HED-Net soft voting strategy consistently enhances accuracy and AUC compared to its individual backbones, demonstrating that the three architectures offer genuinely complementary representations. The confusion matrices and ROC curves in Figures 7–12 demonstrate that ensembling diminishes both false negatives and false positives compared to individual CNNs, which is essential in the context of breast cancer screening.

Statistical investigation with FDR correction indicated that Soft Voting yields genuine performance enhancements, demonstrating considerable improvement over EfficientNetB7 (p < 0.05) and notable trends of enhancement over other individual models, confirmed by consistently superior probability calibration. The HED-Net ensemble has approximately 99 million parameters and necessitates around 0.198 billion FLOPs per inference, in contrast to EfficientNetB7, which contains 64 million parameters and requires approximately 0.128 billion FLOPs. This signifies a considerable rise in complexity (about 55% more FLOPs), although it remains computationally viable for real-time clinical application on contemporary GPUs. The enhancements in diagnostic precision and reliability justify this additional expense, especially in environments with adequate computational capabilities.

The results obtained for the models on the BUSI dataset are given in Table 6, and the results obtained on the BUS-UCLM dataset are given in Table 7. Table 8 shows the performance analysis of various models on the UDIAT dataset.

The ROC curve and confusion matrix obtained for EfficientNetB7, DenseNet121, and ConvNeXtTiny on the BUSI dataset are shown in Figure 9. The ROC curve and confusion matrix obtained for XGBoost and Soft Voting ensemble on the BUSI dataset are shown in Figure 10.

The ROC curve and confusion matrix obtained for EfficientNetB7, DenseNet121, and ConvNeXtTiny on the UDIAT dataset are shown in Figure 11. The ROC curve and confusion matrix obtained for XGBoost and Soft Voting ensemble on the UDIAT dataset are shown in Figure 12.

### 4.6 Visualization using SHAP

**Figure 13:**
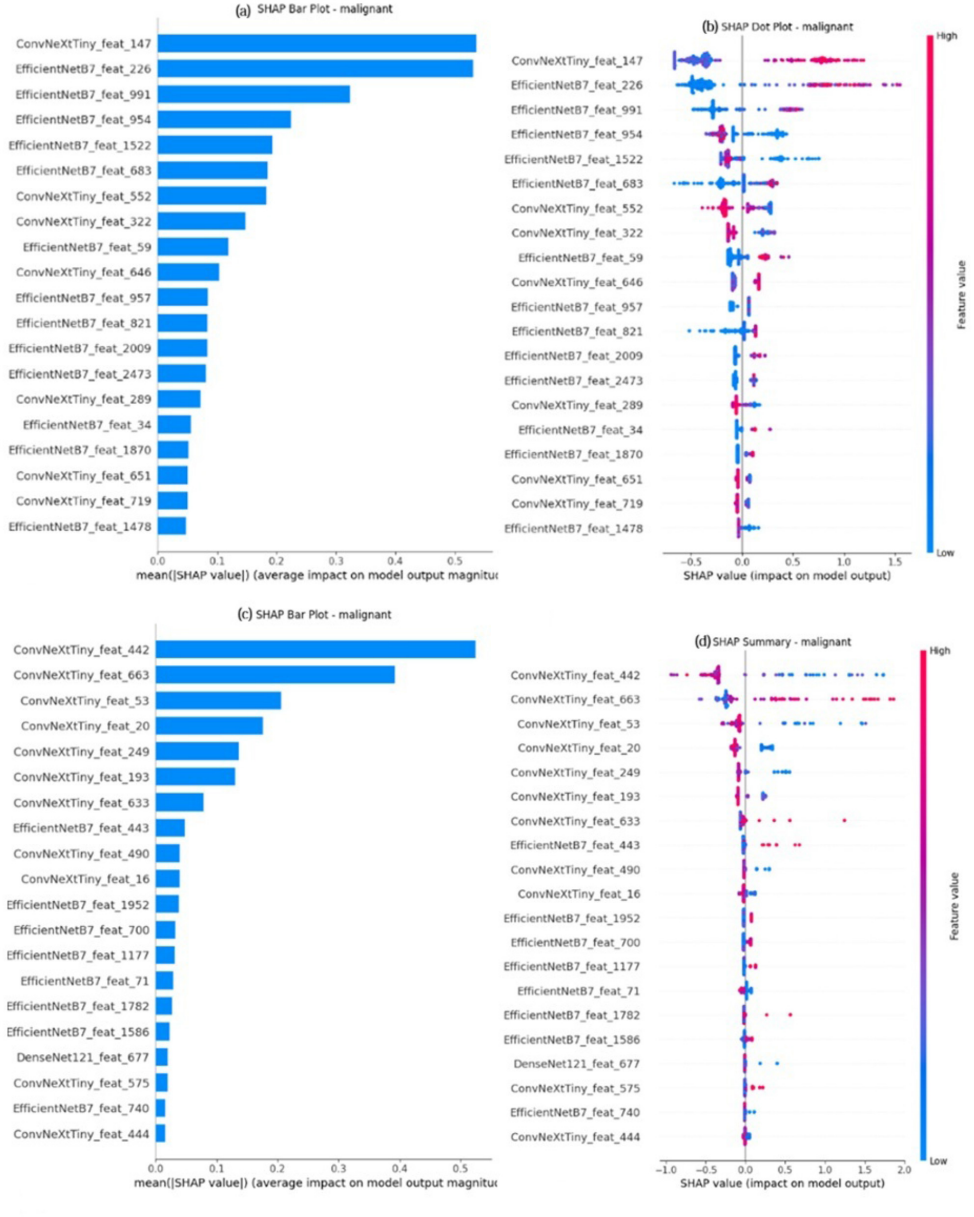
SHAP analysis for the malignant classification on two different datasets. Plots **(a)** and **(c)** are SHAP bar plots for the BUSI and BUS-UCLM datasets, respectively, which rank the top 20 features based on their average impact on the model's output. Plots **(b)** and **(d)** are the corresponding SHAP summary (dot) plots, which illustrate both the direction and magnitude of a feature's effect. Each dot represents a sample, with its horizontal position showing the SHAP value and its color representing the feature's value.

**Figure 14:**
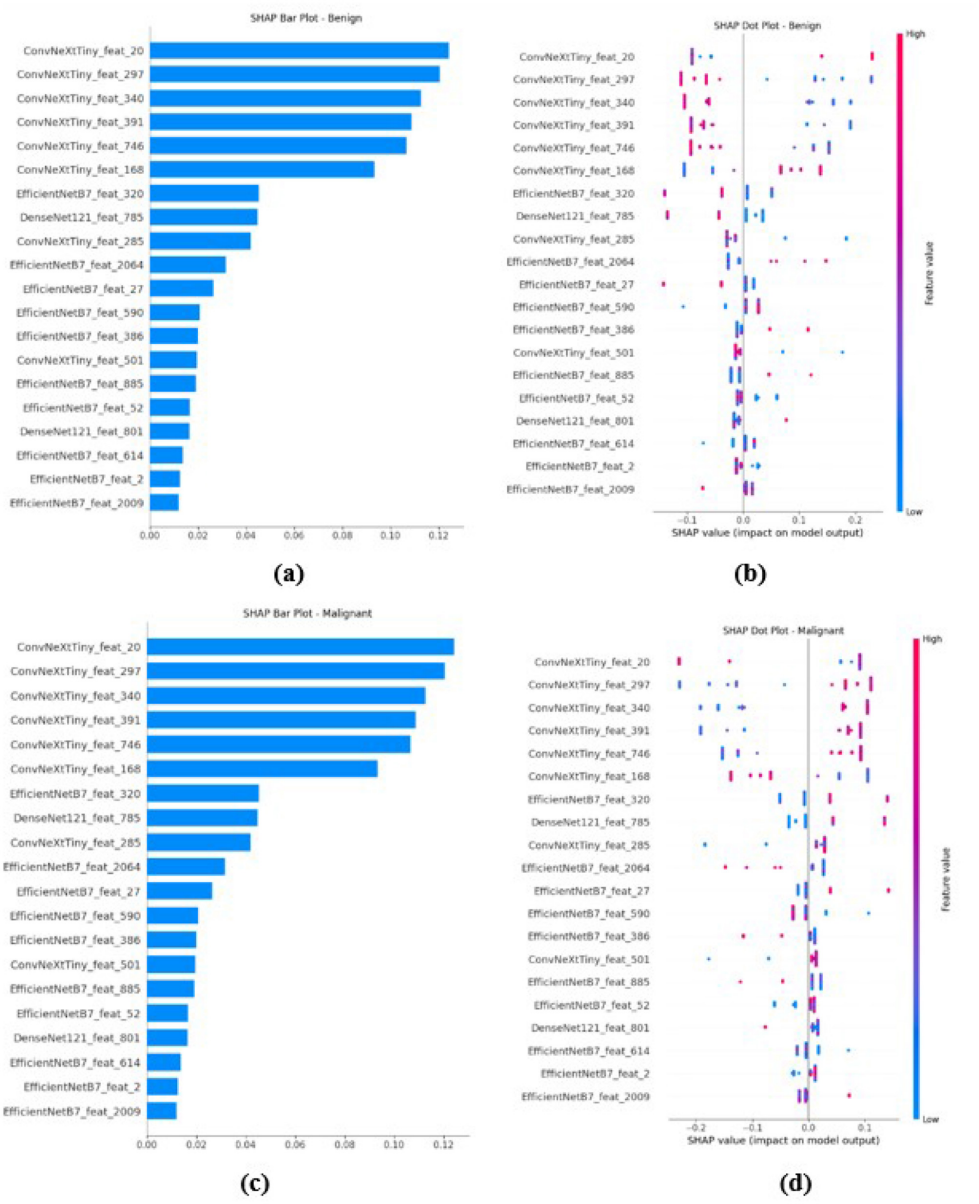
SHAP analysis for the benign and malignant classification on the UDIAT dataset. Plots **(a)** and **(c)** are SHAP bar plots for the benign class, which rank the top 20 features based on their average impact on the model's output. Plots **(b)** and **(d)** are the corresponding SHAP summary (dot) plots, which illustrate both the direction and magnitude of a feature's effect. Each dot represents a sample, with its horizontal position showing the SHAP value and its color representing the feature's value.

SHAP (Shapley Additive exPlanations) analysis is employed to improve the interpretability of the model by assessing the contribution of each feature to the final prediction. The deep features extracted from EfficientNetB7, DenseNet121, and ConvNeXtTiny are concatenated to a singular 4352 dimensional vector and are fed to the XGBoost Classifier. SHAP values are calculated utilizing a unified explainer developed over the trained XGBoost ensemble method. The values are utilized to generate bar plots as well as dot plots for benign, malignant, and normal classes, highlighting the most significant feature per class and its influence on predictions. The SHAP plot for malignant on the BUSI dataset and BUS-UCLM is shown in Figure 13. The SHAP plot for benign and malignant images on the UDIAT dataset is shown in Figure 14.

### 4.7 Visualization using Grad-CAM

**Figure 15:**
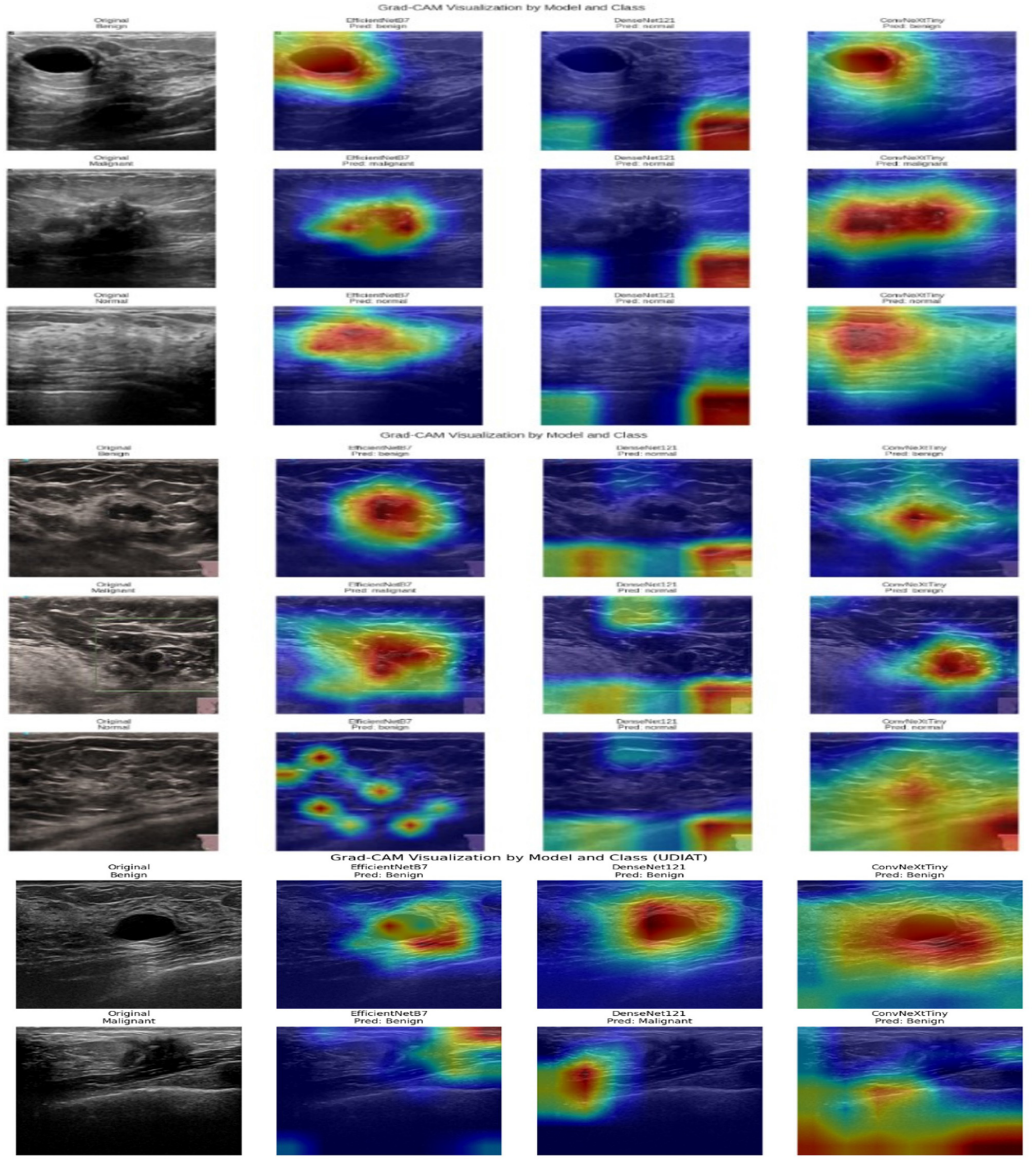
Grad-CAM visualization on benign, malignant, and normal images on **(Top three)** BUSI, **(Middle three)** BUS-UCLM, and **(Bottom two)** UDIAT datasets. Column 1 presents the actual ultrasound images; subsequent columns exhibit the equivalent heatmaps produced by EfficientNetB7, DenseNet121, and ConvNeXtTiny, respectively. Warmer colors like red and yellow signify areas of significant model attention that most substantially influenced the categorization decision, while cooler colors like blue denote minimal contribution.

The Grad-CAM model demonstrates the visual interpretability of the proposed HED-Net model by emphasizing the areas of breast ultrasound images that significantly impacted each model's prediction. Grad-CAM is employed on the last convolutional layer of EfficientNetB7, DenseNet121, and ConvexNet models. The gradients of the predicted class score in relation to the feature maps are calculated using representative test images for the benign, malignant, and normal classes. These maps are then weighted using the averaged gradients to create a heat map. The generated heat maps are superimposed on the original images to indicate the areas of focus for each model during decision-making. The explainable model improves the model transparency and dependability by verifying whether the predictions are based on pertinent tumor regions. The GRAD-CAM visualizations for the benign, malignant, and normal classes on the BUSI dataset are shown in the top three rows of Figure 15, and for the BUS-UCLM dataset are shown in the middle three rows of the Figure 15. The GRAD-CAM visualizations for the benign and malignant classes on the UDIAT dataset are shown in the bottom two rows of Figure 15.

### 4.8 A comparison of the state of the art architectures with the HED-NET architecture

Table 9 delineates the performance of HED-Net compared to notable state-of-the-art methodologies on breast ultrasound datasets. Recent methodologies utilizing singular, extensively optimized architectures or advanced transformer-based frameworks on the BUSI dataset demonstrate exceptionally high accuracies, frequently exceeding 97%. Conversely, HED-Net emphasizes robustness across various datasets and delivers competitive accuracy, attaining a notable AUC of 95.38%. In comparison to transfer learning methods that lack ensemble strategies, HED-Net enhances AUC by several percentage points and provides a more equitable balance between accuracy and probabilistic calibration.

**Table 9:** Comparison of the state-of-the-art architectures with the HED-Net architecture.

**References**	**Dataset**	**Accuracy(%)**	**Precision(%)**	**Recall(%)**	**F1 Score(%)**	**AUC**
Gupta et al. (2023)	BUSI	97.8	99.21	97.68	98.44	–
Deb and Jha (2023)	BUSI	85.23 ± 2.52	–	–	–	–
Nasiri-Sarvi et al. (2024)	BUSI	88.55 ± 1.67	–	–	–	95.28 ± 1.89
	Dataset B	87.50 ± 12.08	–	–	–	92.66 ± 9.07
Alotaibi et al. (2023)	BUSI	87.8	80.8	83.8	83.8	0.9463
	KAIMRC with 5693 images	85.2	75.8	76.4	76	0.9
Meng et al. (2024)	BUSI	98.70	98.80	98.70	98.72	99.82
Jabeen et al. (2022)	BUSI	99.1	99.1		99.08	–
Yadav et al. (2024)	BUSI	97.43	98.55	96.77	97.56	-
Wei et al. (2024)	BUSI	95.0	98.6	91.5	94.9	98.2
	MIBUS	87.4	89.3	91.8	90.6	88.7
Ayana et al. (2022)	Mendeley dataset	99%	-	100	98.9	99.9
	MT small dataset	98.7 ± 1.1%		97.4	96.6	98
Islam et al. (2024)	BUSI	87.82	87.33	85.33	86	–
	UDAIT	85.69	84	78	79.39	–
Dar and Ganivada (2024)	BUSI	96.53	96.59	96.54	96.53	–
	UDIAT	97.51	100	90.54	95.87	–
Kalafi et al. (2021)	Dataset B (163 images) combined with dataset obtained from UMMC (276 images)	93	92	96	94	–
Rezazadeh et al. (2022)	BUSI	91	94	93	93	93
Moon et al. (2020)	BUSI	94.62	90	92.31	91.14	97.11
	SNUH	91.10	90	85.14	89.36	96.97
HED-Net	BUSI	88.46	88.49	88.46	88.45	95.38
	BUS-UCLM	90.51	90.56	90.51	90.51	97.23
	UDIAT	96.97	100	90.91	95.24	99.17

In the BUS-UCLM dataset, which is infrequently utilized in prior studies, HED-Net attains an accuracy of 90.51% and an AUC of 97.23%. This signifies a distinct enhancement over the baseline backbones and illustrates that the ensemble retains its superiority when transitioning from a commonly utilized benchmark (BUSI) to a more contemporary clinical dataset with varying acquisition attributes.

On the UDIAT dataset, HED-Net achieves an accuracy nearing the highest reported methods (exceeding 96%) while concurrently delivering an exceptional AUC of 99.17%. The relative increase in AUC compared to our most robust individual backbone on UDIAT is approximately 2.6%, highlighting that the ensemble enhances not only difficult classification decisions but also confidence calibration, which is particularly critical in borderline or visually ambiguous scenarios.

Although some specialized architectures may attain marginally superior peak accuracies on individual datasets, HED-Net demonstrates consistent and well-calibrated performance across three distinct public datasets, yielding relative enhancements over its constituent backbones of approximately 2% to nearly 7% in accuracy and up to about 2.6% in AUC. When integrated with Grad-CAM and SHAP-based explanations, HED-Net emerges as a robust and interpretable option for implementation in various clinical settings.

## 5 Discussion

The HED-Net framework illustrates the effectiveness of hybrid ensemble learning for classifying breast ultrasound images across diverse datasets with differing characteristics. The performance analysis uncovers several critical insights concerning model design, generalization ability, and clinical relevance.

### 5.1 Ensemble effectiveness and complementary feature extraction

The enhanced efficacy of the soft voting ensemble compared to individual models highlights the importance of integrating complementary architectures. EfficientNetB7, utilizing compound scaling and depthwise separable convolutions, effectively captures intricate local textures, which is particularly advantageous for differentiating subtle morphological variations between benign and malignant lesions. The dense connectivity of DenseNet121 enabled hierarchical feature reuse, maintaining structural details and edge continuity essential for analyzing lesion boundaries. ConvNeXtTiny, influenced by transformer architectures, offered strong global context modeling via large-kernel operations, improving sensitivity to spatial relationships within the ultrasound image. The 2.2–6.7% enhancements in accuracy of soft voting compared to the optimal single model across datasets validate that ensemble diversity diminishes variance and alleviates individual model biases.

### 5.2 Generalization across datasets

The consistent performance of HED-Net across the BUSI, BUS-UCLM, and UDIAT datasets underscores its generalization ability. The model attained accuracies of 88.46%, 90.51%, and 96.97%, respectively, illustrating its adaptability to differences in image acquisition protocols, ultrasound machine models, and patient demographics. The BUS-UCLM dataset, characterized by its superior resolution and intricate segmentation masks, enabled the model to utilize structural annotations during training, yielding robust performance despite its reduced size. On the UDIAT dataset comprising solely benign and malignant classes, HED-Net achieved an AUC of 99.17%, demonstrating exceptional discriminative capability for binary classification tasks.

### 5.3 Interpretability and clinical trust

The black box nature of deep learning models is a major obstacle to clinical adoption that is addressed by the integration of SHAP and Grad-CAM visualizations. Grad-CAM heatmaps consistently emphasized clinically significant regions. SHAP analysis indicated that features from EfficientNetB7 and ConvNeXtTiny were predominantly influential in malignant classification, whereas DenseNet121 features were more significant for benign cases. This indicates that malignant lesions are more accurately defined by local texture irregularities and global spatial deformations, while benign lesions display more structured patterns.

## 6 Conclusion and future scope

A hybrid ensemble deep learning framework has been put forth to accurately classify breast ultrasound images into three categories: normal, malignant, and benign. The design integrates EfficientNetB7, DenseNet121, and ConvNeXtTiny to leverage the complementary characteristics of various architectures. EfficientNetB7 has superior computing efficiency and local pattern extraction owing to its compound scaling and depthwise convolutions. DenseNet121 enhances fine-grained feature learning by its dense connectivity, facilitating feature reuse and enhancing gradient flow. ConvNeXtTiny enhances architectural diversity by utilizing larger kernel sizes to capture extensive spatial relationships and employing contemporary convolutional designs. Two ensemble methodologies, feature-level fusion utilizing XGBoost and soft voting at the output stage, were employed for classification. The feature-level ensemble method attained an accuracy of 87.19% on BUSI and 86.13% on BUS-UCLM, whereas the soft voting ensemble method enhanced the results to 88.46% and 90.51%, respectively. To improve interpretability, SHAP and Grad-CAM approaches were utilized, offering insight into the decision-making process of the model and emphasizing clinically significant tumor locations.

The HED-Net model, even though it exhibits great results, has numerous drawbacks that require attention. The dependence on publicly accessible datasets imposes limitations on sample size, demographic variety, and imaging variability. The datasets employed in this investigation are very small, and the class imbalance can impact the model's sensitivity and potentially diminish recall for clinically ambiguous lesions. The utilization of numerous datasets aids in evaluating cross-dataset resilience, but the images are derived from certain scanners, geographic areas, and clinical methodologies, which may not adequately represent the heterogeneity found in extensive multi-institutional environments. The HED-Net ensemble elevates computing demands owing to the incorporation of three backbone networks and an additional fusion layer.

Future research will concentrate on verifying HED-Net using larger, multi-institutional datasets to guarantee scalability and clinical dependability. In addition, expanding HED-Net to accommodate various imaging modalities and tumor types will further enhance its generalization across datasets. Furthermore, augmenting the interpretability aspect with multimodal explainable AI and clinician in the loop validation may boost clinical trust and promote real-world implementation of computer-aided diagnostic systems.

## Data Availability

The original contributions presented in the study are included in the article/supplementary material, further inquiries can be directed to the corresponding author.
